# Endurance exercise elicits temporal and sexual dimorphic multi-omics remodeling of liver metabolism revealed by MoTrPAC

**DOI:** 10.1016/j.celrep.2026.117416

**Published:** 2026-06-08

**Authors:** Taylor J. Kelty, Edziu Franczak, Nicole R. Gay, Gina M. Many, Tyler J. Sagendorf, James A. Sanford, Zhenxin Hou, David A. Gaul, Facundo M. Fernández, Michaella Rekowski, Charles F. Burant, Andrea L. Hevener, Joshua N. Adkins, Sue Bodine, Malene E. Lindholm, Eric A. Ortlund, Simon Schenk, John P. Thyfault, R. Scott Rector, MoTrPAC study group

**Affiliations:** 1 NextGen Precision Health, University of Missouri-Columbia, Columbia, MO, USA; 2 Department of Nutrition and Exercise Physiology, University of Missouri, Columbia, MO, USA; 3 Division of Gastroenterology and Hepatology, Department of Medicine, University of Missouri, Columbia, MO, USA; 4 Research Service, Harry S. Truman Memorial VA Medical Center, Columbia, MO, USA; 5 Departments of Cell Biology and Physiology and Internal Medicine, University of Kansas Medical Center, Kansas City, KS, USA; 6 Kansas Center for Metabolism and Obesity Research and KU Diabetes Institute, University of Kansas Medical Center, Kansas City, KS, USA; 7 Department of Genetics, Stanford University, Stanford, CA, USA; 8 Biological Sciences Division, Pacific Northwest National Laboratory, Richland, WA, USA; 9 Department of Biochemistry, Emory University School of Medicine, Atlanta, GA, USA; 10 School of Chemistry and Biochemistry, Georgia Institute of Technology, Atlanta, GA, USA; 11 Department of Cancer Biology, University of Kansas Medical Center, Kansas City, KS, USA; 12 Mass Spectrometry and Proteomics Core Facility, University of Kansas Medical Center, Kansas City, KS, USA; 13 Division of Metabolism, Endocrinology, and Diabetes, Department of Internal Medicine, University of Michigan, Ann Arbor, MI, USA; 14 Division of Endocrinology, Diabetes, and Hypertension, Department of Medicine, University of California, Los Angeles, CA, USA; 15 Aging and Metabolism Research Program, Oklahoma Medical Research Foundation, Oklahoma City, OK, USA; 16 Division of Cardiovascular Medicine, Department of Medicine, Stanford University, Stanford, CA, USA; 17 Department of Orthopedic Surgery, School of Medicine, University of California, San Diego, La Jolla, CA, USA; 18 Research Service, Kansas City VA Medical Center, Kansas City, MO, USA; 19 Senior author; 20 These authors contributed equally; 21 Co-first authors; 22 Lead contact

## Abstract

The mechanisms by which exercise modulates liver metabolism are poorly understood. Leveraging data from molecular transducers of physical activity consortium (MoTrPAC), we analyzed liver adaptations across 1, 2, 4, and 8 weeks of exercise in male and female rats using multi-omics approaches. Female livers displayed a progressive increase in oxidative phosphorylation (OXPHOS) protein complexes, while male livers showed an increased acetylation of OXPHOS, tricarboxylic acid cycle, and fatty acid oxidation enzymes. Mechanistic examination revealed that these sex-specific acetylation events are partially mediated by carnitine acetyltransferase. Exercise enhanced liver cholesterol and bile acid synthesis, reducing liver lipid metabolites in males after 8 weeks of exercise. Male rats had higher fecal cholesterol and cholic acid levels, indicating a sex-specific mechanism of lipid excretion with exercise. Eight weeks of training reduced markers related to hepatic stellate cell activation and fibrosis in both sexes. This study highlights the sexual dimorphic and temporal molecular signatures by which exercise modulates liver metabolism to provide hepatoprotective effects.

## In brief

Endurance training affects male and female livers differently, with males increasing mitochondrial protein function and females increasing mitochondrial proteins. Exercise also increased the liver’s capacity for bile acid and cholesterol synthesis; however, the timing of the response differed, with males increasing fecal lipid excretion after chronic exercise training.

## Graphical abstract

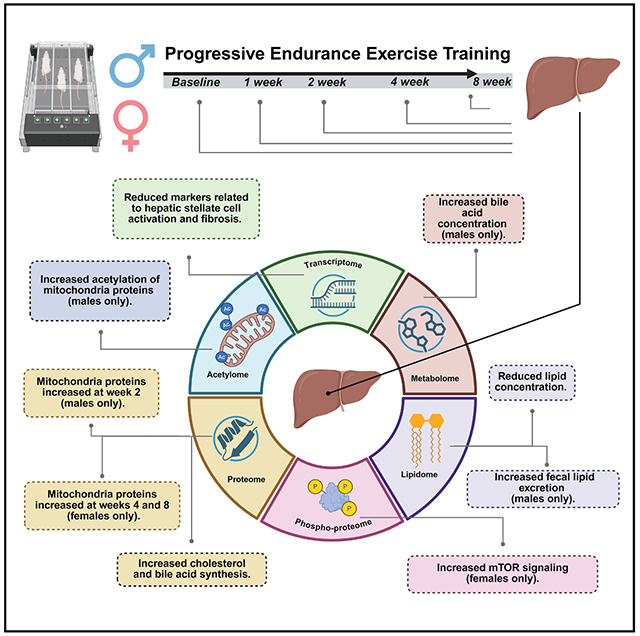

## INTRODUCTION

Cardiorespiratory fitness is the most significant predictor for all-cause mortality in both men and women.^1,2^ Endurance exercise has been shown to improve parameters of metabolic health,^3^ particularly in patients with metabolic syndromes,^4,5^ serving as an accessible frontline defense against metabolic diseases, including metabolic-dysfunction-associated steatotic liver disease (MASLD),^6–8^ colloquially known as fatty liver disease. During periods of metabolic stress (i.e., exercise and fasting), the liver modulates its uptake (fatty acids, lactate, amino acids, etc.) and secretion (glucose and ketones) of metabolites in response to metabolic and endocrine signaling to meet systemic energetic needs.^9,10^ Importantly, exercise training imparts a durable liver adaptation, promoting tighter coupling of ATP synthesis to glucose production at any given workload.^11–13^ Because the liver is critical in orchestrating substrate availability to tissues during exercise and is central to metabolic regulation, understanding exercise-induced molecular adaptations in the liver is critically important.^14–16^

Rodent studies from the last half century have revealed a myriad of exercise-induced liver adaptations ranging from markers of elevated mitochondrial function^12,17,18^ to robust changes in glucose metabolism.^10,19–21^ Further research has corroborated and enhanced these findings to reveal that exercise can effectively prevent and reverse MASLD in both preclinical models and humans.^22–28^ Omics-based approaches have recently been employed to better understand exercise-induced liver adaptations in transcripts,^25^ proteins,^26,29–31^ and metabolites.^27,28^ Moreover, a substantial proportion of studies have focused on the male sex, despite evidence of a sexual dimorphism in liver mitochondrial dynamics.^11,13,32–34^ Here, this study applied multi-omics analyses to capture temporal liver adaptations to exercise.

The purpose of the molecular transducers of physical activity consortium (MoTrPAC)^35^ is to provide detailed maps of the multi-omics response to acute physical activity and exercise training across tissues.^36^ We have recently published findings on the multi-omics changes across tissues in exercise-trained (1, 2, 4, or 8 weeks of treadmill running) female and male rats (aged 6 months).^35,37^ Rats adequately reflect human responses to exercise in blood biochemical profiles and mitochondrial adaptations by 8 weeks,^38,39^ conceptually comparable to 4–6 months of sustained training in humans. In this cohort of rats, we characterized the temporal training response across mitochondrial analytes,^40^ transcriptomic and epigenetic signatures,^41^ complex trait genetics,^42^ and adiposity.^43^ These studies demonstrated that the liver exhibited the highest number of significantly enriched metabolite classes,^35^ which were strongly correlated with plasma metabolites, underscoring the liver’s central role in whole-body energy homeostasis.^40^ Endurance training induced pronounced effects on the liver multi-ome, with most of these changes associated with mitochondrial and lipid metabolic pathways^35^; notably, mitochondrial protein acetylation represented over 60% of the significant changes observed in the liver following chronic exercise training.^40^ Collectively, these findings underscored the value of a focused investigation of the liver, which had not yet been undertaken by the MoTrPAC.

Utilizing the data generated by MoTrPAC,^35^ we extended these findings by providing an in-depth analysis of the temporal effect of exercise on the liver multi-ome, with a focus on two of the most enriched pathways revealed: mitochondrial and lipid metabolism. Further extending previous findings from MoTrPAC,^35^ our analyses led us to generate novel fecal metabolome and bile acid (BA) datasets to investigate mechanisms mediating exercise-induced lipid excretion. These findings provide valuable insights into how chronic exercise impacts liver mitochondrial and lipid metabolism, outcomes that can be leveraged to develop treatments that target optimal liver health and prevent or treat MASLD.

## RESULTS

### Sex-specific and temporal liver remodeling is associated with enhanced mitochondrial and lipid metabolism following chronic exercise training

**Figure 1.**
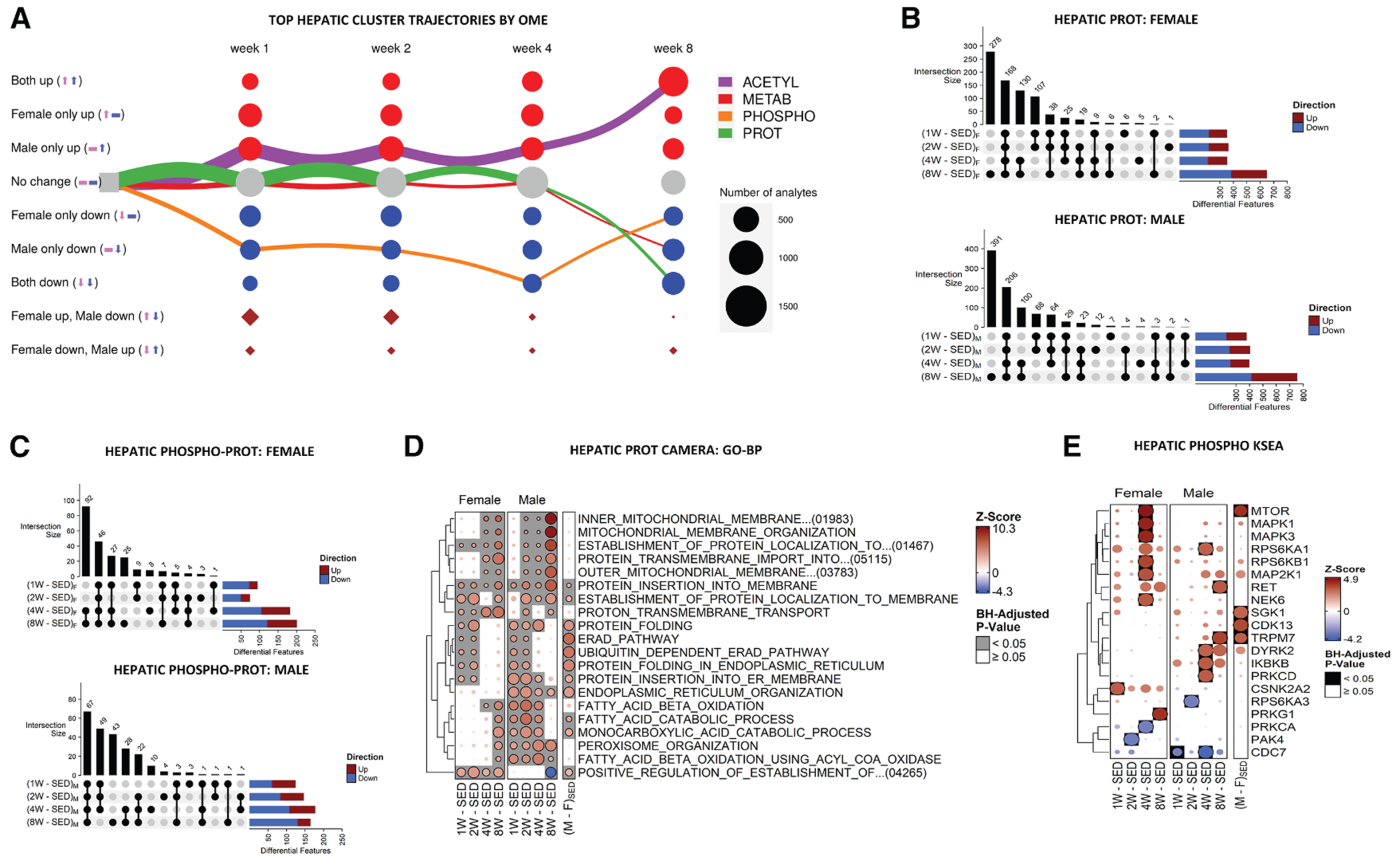
Sex-specific and temporal liver remodeling is associated with enhanced mitochondrial and lipid metabolism following chronic exercise training. (A) Graphical representation of training-differential analytes in liver tissue. Here, features were first filtered in a time point-independent manner as determined by the F-test, and then temporal trajectories for males and females were displayed according to the resultant timewise differential analysis. This graph includes the largest paths, with both node and edge size being proportional to the number of analytes represented. (B and C) Upset plots of statistically significant (FDR < 0.05) proteins (B) and phosphoproteins (C) between age-matched trained and sedentary females and males. (D) Bubble heatmaps depicting top biological processes (BP) from the gene ontology (GO) database across training and sedentary conditions. Heatmaps were derived from the proteomic CAMERA-PR results. Circles are colored according to the set-level *Z* score and scaled by row so that the most significant comparison is of maximum area. (E) Inferred activity of the indicated kinases across 8 weeks of training in liver tissue. Blue bubbles indicate a decrease, and red indicate an increase in endurance training compared to sedentary rats or a decrease in sedentary male compared to sedentary female rats. A gray or black background indicates a significant result (adjusted *p* value < 0.05, *n* = 5/sex/condition).

To identify the most prominent temporal or sex-associated liver exercise adaptations, we first examined each of the omes in response to progressive exercise training (Figure 1A). Longitudinal differential analyte trajectories were identified using a graphical clustering approach applied to timewise differential analysis across the exercise intervention, rather than a single two-group comparison. This approach allowed identification of analytes that behave similarly at one point, then diverge or re-align at later time points. Edges are split by each of the top 4 impacted omes analyzed (unable to identify a transcript trajectory due to relatively limited transcript adaptations across time), where each node represents 1 of 9 states (row labels) at each of the 4 sampled training time points (column labels). Edges are drawn between adjacent nodes to represent the path of differential analytes over the training time course.

Exercise training induced distinct temporal patterns between differentially regulated proteins, representing combined 794 (female) and 914 (male) differentially abundant proteins (DAPs) and 227 (female) and 233 (male) phosphosites across all time points in false discovery rate (FDR) < 0.05; Figures 1B and 1C). To bridge our understanding between differentially expressed analytes and related biological functions, we performed preranked correlation-adjusted mean rank gene set testing (CAM-ERA-PR) to test for enrichment of Gene Ontology (GO) biological processes (BP)^44–48^ (Figures 1D and 1E).

Female and male liver proteomes were enriched for terms associated with mitochondrial metabolic pathways with chronic exercise training (mitochondrial membrane organization and fatty-acid beta oxidation), with the male liver acquiring these adaptations earlier (week 2 of training) compared to females who acquired them later (week 4 of training) (Figure 1D). The slower adaptation in females is likely attributable to their displaying enhanced mitochondrial oxidative phosphorylation (OXPHOS) profiles in the sedentary condition compared to males. CAMERA-PR was also applied to sets of phosphosites grouped according to their known kinases to examine changes in predicted kinase activity (Figure 1E). We refer to the use of CAMERA-PR in conjunction with kinase sets as kinase-substrate enrichment analysis (KSEA).

### Chronic exercise altered liver transcriptome to promote sterol metabolism in hepatocytes, inhibiting HSC activation independent of sex

**Figure 2.**
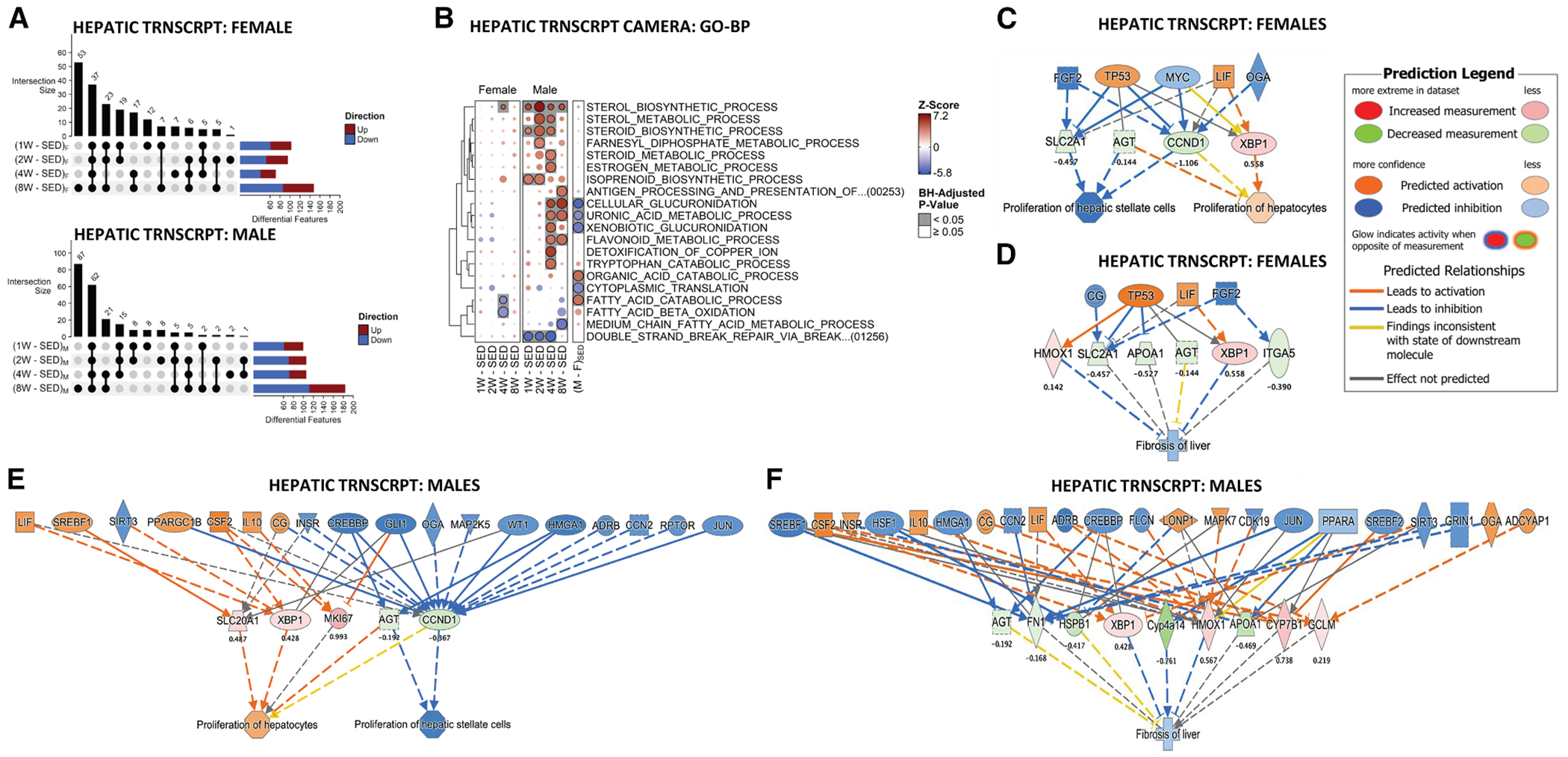
Chronic exercise altered liver transcriptome to promote sterol metabolism in hepatocytes, inhibiting hepatic stellate cell activation independent of sex. (A) Upset plots of statistically significant (FDR < 0.05) transcripts between age-matched trained and sedentary females and males. (B) Bubble heatmap depicting top biological processes (BP) from the gene ontology (GO) database across training and sedentary conditions. The heatmap was derived from the transcriptomic datasets using the CAMERA-PR method. Blue bubbles indicate a decrease and red an increase in endurance training compared to sedentary rats or a decrease in sedentary male compared to sedentary female rats. A gray or black background indicates a significant result (adjusted *p* value < 0.05). (C and D) Predicted decreased proliferation of stellate cells and increased proliferation of hepatocytes (C) and a decrease in fibrosis (D) of the liver after 8 weeks of treadmill running in females. (E and F) Decreased proliferation of stellate cells and increased proliferation of hepatocytes (E) and a decrease in fibrosis (F) of the liver after 8 weeks of treadmill running in males. DEGs take all time points and sexes into account (FDR < 0.05). Blue and green colors represent a decrease, and orange and red colors represent an increase in endurance training compared to sedentary rats (*n* = 5/sex/condition). Expression levels of DEGs are shown below each node and displayed as log2FC (fold change). DEGs with *Z* score > 2 were considered activated.

Exercise training-induced distinct temporal patterns between differentially regulated transcripts, representing a combination of 192 and 226 downregulated differentially expressed genes (DEGs) across all time points in females and males, respectively (FDR < 0.05, Figure 2A). Across 8 weeks of exercise training, the male liver transcriptome was positively enriched for terms related to sterol biosynthetic and metabolic processes (Figure 2B). Sterol biosynthetic processes were also enriched for the females but were not observed until after 4 weeks of exercise training (Figure 2B). Ingenuity pathway analysis (IPA) was used to generate mechanistic networks based on top-predicted biological functions and upstream regulators following 1, 2, 4, or 8 weeks of exercise training. IPA predicted decreased hepatic stellate cell (HSC) activation and fibrosis with increased hepatocyte proliferation, independent of sex (Figures 2C–2F). Markers of reduced HSC activation and fibrosis revealed by identified DEGs included angiotensinogen (AGT) and cyclin D1 (CCND1). AGT and CCND1 can indirectly promote fibrosis^,50^ and activation of HSCs.^51^

### Female-specific liver proteome upregulation of OXPHOS complexes, carbohydrate metabolism, and amino acid metabolism with chronic exercise training

**Figure 3.**
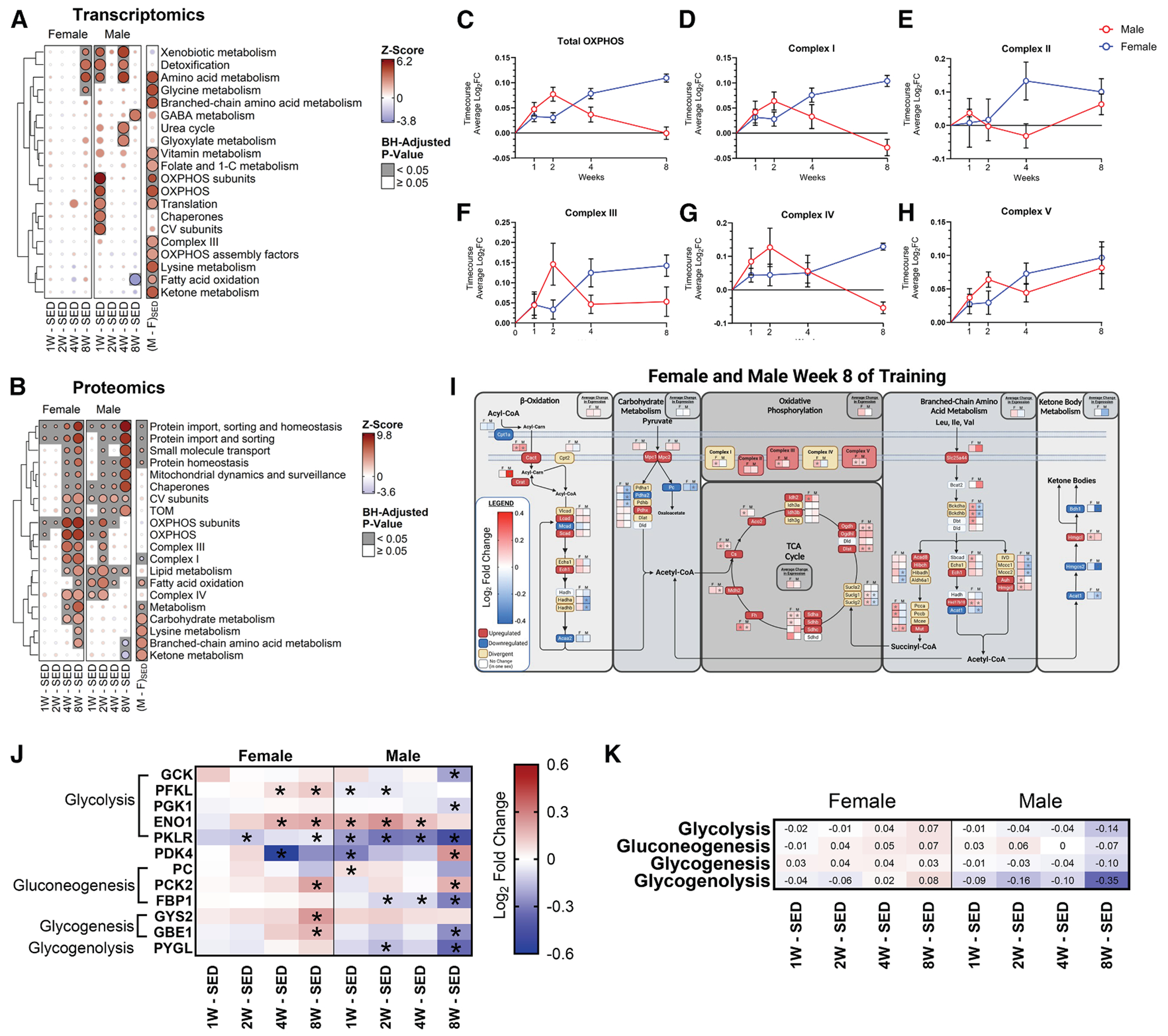
Female-specific liver proteome increases in abundance of OXPHOS complexes, carbohydrate metabolism, and amino acid metabolism with chronic exercise training. (A and B) Bubble heatmaps indicating the direction and magnitude of change in abundance of the top 20 most significantly altered pathways within the (A) transcriptome and (B) proteomic differences following exercise training and at sedentary levels. (C) Time course training effect on average log2FC protein abundance of all proteins involved in OXPHOS. (D–H) Change in average protein log2FC abundance during the 1, 2, 4, and 8 weeks of training for each complex of OXPHOS. (I) Log2FC in protein abundance at 8 weeks of training for all significantly altered metabolic processes within the liver mitochondria generated via BioRender. (J and K) Time course training effect on individual and average log2FC protein abundance of all proteins involved in carbohydrate metabolism. Log2FC Protein labels in red represent increases in abundance, and blue represent decreases in abundance within both male and female rats (*n* = 5/sex/condition). Yellow-labeled proteins show divergent change in the direction of abundance between males and females. White labels represent no change in abundance within at least one sex (log2FC < 0.01). Significant protein abundance change compared to sedentary conditions within sex is presented by *p < 0.05. Values are represented as mean ± standard error.

Proteomic enrichment revealed mitochondrial adaptations to chronic exercise, with MitoCarta3.0 analyses showing modest transcript-level changes (Figure 3A) but robust increases in mitochondrial metabolic proteins (Figure 3B). While total mitochondrial protein content was similar between sedentary males and females (Figure S1A), sedentary males displayed higher abundance of proteins involved in fatty acid oxidation (FAO), carbohydrate, ketone, and branched-chain amino acid metabolism (*p* < 0.05, Figure 3B), whereas females exhibited higher complex I OXPHOS abundance (*p* < 0.05). Exercise induced a sustained increase in mitochondrial protein abundance across multiple metabolic pathways in females (*p* < 0.05; Figure 3B), while males showed only transient increases in FAO and OXPHOS proteins during the first 2 weeks (*p* < 0.05). Both sexes demonstrated increased mitochondrial protein import across all training phases (*p* < 0.05), and exercise-induced proteomic changes were not reflected at the transcript level (Figure 3A), likely due to rapid hepatic mRNA turnover following exercise cessation.^52^

Male and female rats showed distinct OXPHOS responses to exercise (Figure 3C). Females exhibited progressive increases across multiple OXPHOS complexes throughout training, resulting in a 7.8% overall increase (Figures 3C–3H). Conversely, males showed transient increases in complexes I, III, IV, and V during the first 2 weeks (*p* < 0.05), with only complex V remaining elevated through 8 weeks (Figures 3D–3H). These changes occurred without any alteration in the expression of mitochondrial biogenesis regulators. Overall, females displayed a more robust proteomic response to exercise, with greater increases in mitochondrial metabolic and OXPHOS proteins (Figure 3I). While most proteins changed similarly between sexes in response to endurance exercise, several (including HADHA/B, PDHA1/B, SUCLG1/2, BCKDHA/B, and OXPHOS complexes I and IV) showed sex-specific divergence, with increased abundance in females but decreased abundance in males.

Although exercise maintains euglycemia through increased hepatic glucose output,^53,54^ training induced limited changes in glycolytic/gluconeogenic and glycogen metabolic proteins (Figures 3J and 3K). In females, only a few enzymes showed increased abundance with prolonged training (PFKL, ENO1, PCK2, GYS2, and GBE1; Figure 3J). In contrast, males exhibited more dynamic responses, with early, sustained, or delayed changes across multiple enzymes (Figures 3J and 3K). Overall, chronic exercise tended to increase these proteins in females but decrease them in males.

### Sex-specific increase in OXPHOS, tricarboxylic acid cycle, and FAO enzymes in male liver acetylome with chronic exercise training

**Figure 4.**
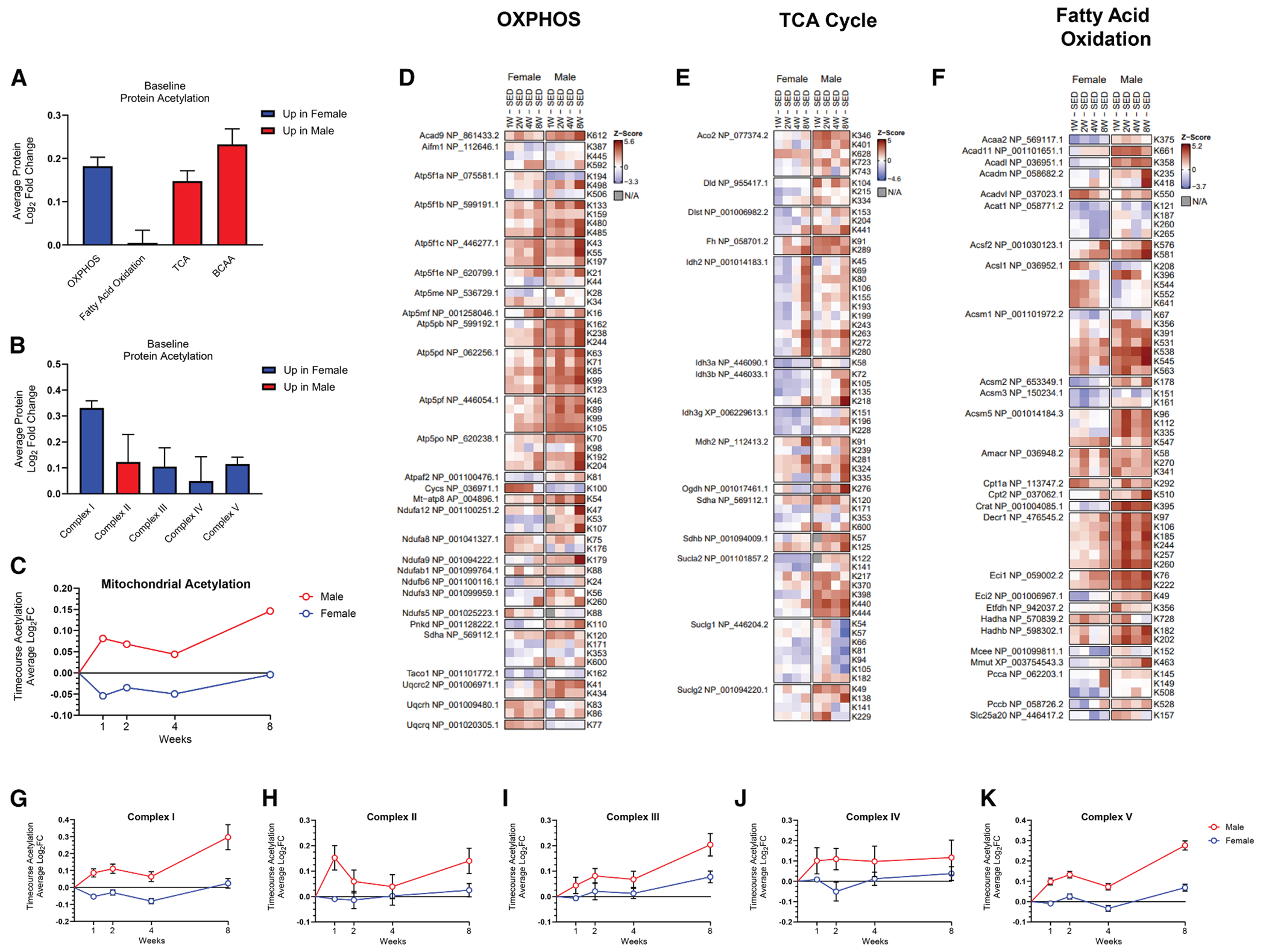
Sex-specific increase in OXPHOS, TCA cycle, and fatty acid oxidation enzymes in male liver acetylome with chronic exercise training. (A) Average log2FC of mitochondrial protein acetylation involved in OXPHOS, FAO, TCA cycle (TCA), and branched chain amino acid catabolism between sedentary male and female rats. (B) Average log2FC acetylation of each OXPHOS complex protein between male and female sedentary rats. (C) Average log2FC acetylation changes across the exercise training paradigm. (D–F) Heatmap showing the change in abundance of the most significantly altered acetylation events during each training time point (1−, 2−, 4−, and 8-week SED) in male and female rats within (D) OXPHOS, (E) TCA cycle, and (F) FAO. (G–K) OXPHOS complex I, complex II, complex III, complex IV, and complex V average log2FC in acetylation events over the course of the training paradigm (*n* = 5/sex/condition). Values are represented as mean ± standard error. For acetylation heatmaps, each contrast was filtered to the top 10 sites with the smallest *p* values after filtering to the genes present in each gene set.

Our previous report demonstrated that exercise increased mitochondrial protein acetylation in the liver, particularly affecting proteins involved in OXPHOS, tricarboxylic acid (TCA) cycle, FAO, and branched-chain amino acid catabolism.^40^ Here, we extended the earlier analysis by examining innate sex differences and the progressive impact of exercise training on liver mitochondrial acetylation. Despite there being limited differences in overall acetylation levels under sedentary conditions between sexes (Figure S1A), protein-specific enrichment for acetylation correlated with sex differences in total protein abundance. In female sedentary rats, there was a 13% greater accumulation of acetylation events on OXPHOS proteins (Figure 4A), whereas males exhibited higher levels of acetylated proteins in the TCA cycle (11% greater) and branched-chain amino acid catabolism pathways (17% greater; Figure 4A). Sedentary female rats demonstrated greater overall acetylation levels across all complexes within OXPHOS, excluding complex II (Figure 4B).

Next, we explored the specific lysine acetylation sites of OXPHOS, the TCA cycle, and FAO proteins. Unlike protein abundance, exercise training promoted progressive accumulation of acetylation events on proteins involved in OXPHOS, TCA cycle, and FAO (Figures 4C–4F and S1C–S1E). The influx of acetylation events on the OXPHOS system was not limited to a particular complex, with all five complexes showing increased aggregate acetylation events throughout training, although complex I and V were particularly susceptible to increased acetylation in males (Figures 4G–4K). In contrast, aggregate acetylation levels for each of these pathways remained stable throughout 8 weeks of exercise training in female rats. Changes in individual complexes’ acetylation status mimic alterations in aggregated acetylation events within the mitochondria of both male and female rats after training (Figure 4C).

### CrAT in the liver controls mitochondrial acetylation corresponding to the impact of exercise in male livers

**Figure 5.**
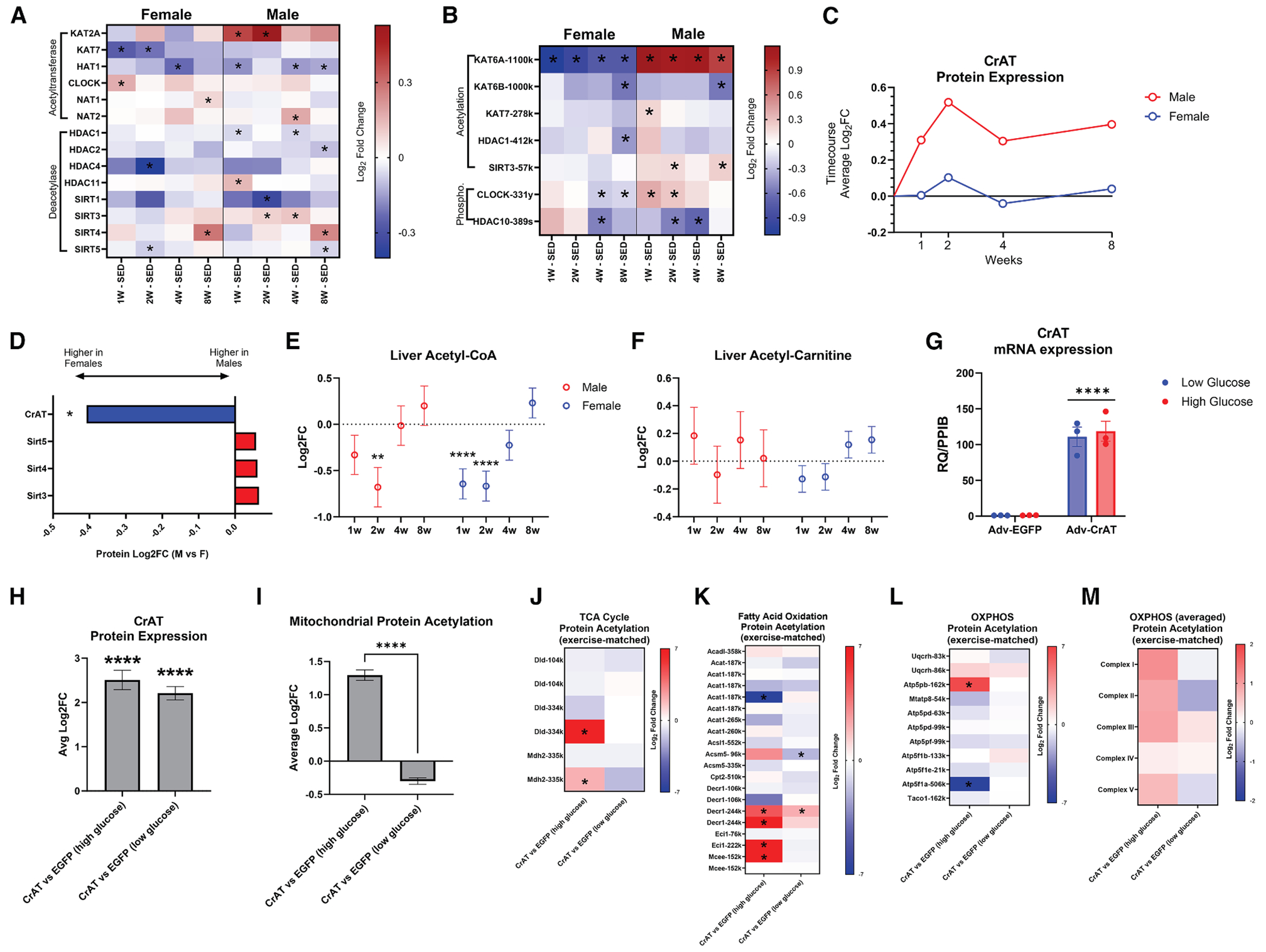
CrAT in the liver regulates mitochondrial acetylation corresponding with exercise-induced changes in males. (A and B) Log2FC of deacetylases and acetyltransferases (A) individual enzyme expression (B) and post-translational modification across the exercise training paradigm. (B) CrAT protein expression over the exercise training paradigm. (C) Log2FC of deacetylases and acetyltransferase enzymes between sedentary male and female rats. (E and F) LogFC of liver metabolites (E) acetyl-CoA and (F) acetyl-carnitine across exercise training paradigm. (G) Gene expression of CrAT in primary mouse hepatocytes. (H) Log2FC protein expression of CrAT in primary hepatocytes with high and low glucose concentrations. (I) Log2FC of aggregated mitochondrial protein acetylated events in primary hepatocytes. (J and K) Heatmap showing the log2FC in individual acetylation events (corresponding with exercise acetylation events) in primary hepatocytes within the (J) TCA cycle, (K) FAO, and (L) OXPHOS. (M) Averaged OXPHOS complex (I–V) acetylation in primary hepatocytes. For acetylation heatmaps, each contrast was filtered to the individual acetylation event that changed with exercise training in Figure 4. Values are represented as mean ± standard error. *Indicates signifiance, *p < 0.05, **p < 0.01, ****p < 0.0001.

To identify drivers of acetylation in males, we examined acetyltransferase and deacetylase expression (Figure 5A). Training induced minimal changes, with increased KAT2A and NAT3, reduced HDAC1/2, SIRT1, and SIRT5, and increased mitochondrial sirtuins SIRT3 and SIRT4. Post-translational modification (PTM) changes were limited to only KAT6A and KAT6B. Protein abundance of the mitochondrial sirtuins (SIRT3 and SIRT4) increased with training, as previously reported^40^ (Figure 5B). Given these modest effects, we examined metabolic acetyltransferases and identified a male-specific, training-induced increase in carnitine O-acetyltransferase (CrAT) abundance (Figure 5C), while females displayed higher basal CrAT expression (Figure 5D). Exercise increased hepatic CrAT in males to levels comparable to sedentary females, suggesting sex-dependent mitochondrial acetyl-CoA handling.

Exercise increases glucose flux and hepatic glycogen repletion,^55^ which, together with high CrAT expression, may promote acetylation. Exercise reduced liver acetyl-CoA after 2 weeks in both sexes, normalizing by 4 weeks in males but remaining reduced until 8 weeks in females (Figure 5E), while acetyl-carnitine levels were unchanged (Figure 5F). Stable acetyl-carnitine suggests bidirectional CrAT activity, potentially buffering mitochondrial acetyl-CoA during early training and contributing to increased acetylation in males as an adaptive response. Despite similar reductions in acetyl-CoA, females showed no change in acetylation (Figure 4C), possibly due to higher basal CrAT levels (Figure 5D). To test CrAT function under exercise-like conditions, CrAT was overexpressed in primary hepatocytes, significantly increasing CrAT mRNA and protein under both low- and high-glucose conditions (Figures 5G and 5H). CrAT overexpression increased mitochondrial acetylation under high glucose but modestly reduced it under low glucose (Figure 5I), indicating glucose-dependent, bidirectional regulation of acetylation by CrAT.

CrAT overexpression under high-glucose conditions increased mitochondrial acetylation, which recapitulated exercise-induced changes observed in male livers. Functional categorization revealed increased acetylation of TCA cycle proteins (DLD-334K and MDH2; Figure 5J), FAO proteins (DECR1-244K, MCEE-152K, and ECI1-222K), and decreased ACAT1-187K acetylation (Figure 5K), consistent with exercise training. Under low glucose, CrAT overexpression had minimal effects, limited to two FAO-related acetylation events (ACSM5-96K and DECR1-224K; Figure 5K). Components of complex V were differentially acetylated under high glucose (increased ATP5BP-162K and decreased ATP5F1A-506K; Figure 5L), and aggregated acetylation across OXPHOS complexes I–V was increased only under high-glucose conditions (Figure 5M). Global proteomics revealed reduced OXPHOS protein abundance with CrAT overexpression, particularly under low glucose (Figures S2A–S2J), aligning with male training data showing increased mitochondrial acetylation without robust OXPHOS protein accumulation. A full list of acetylation-related changes can be found in Tables S2, S3, and S4.

### Liver cholesterol and BA biosynthesis are persistently increased in males but temporally delayed in females with chronic exercise training

**Figure 6.**
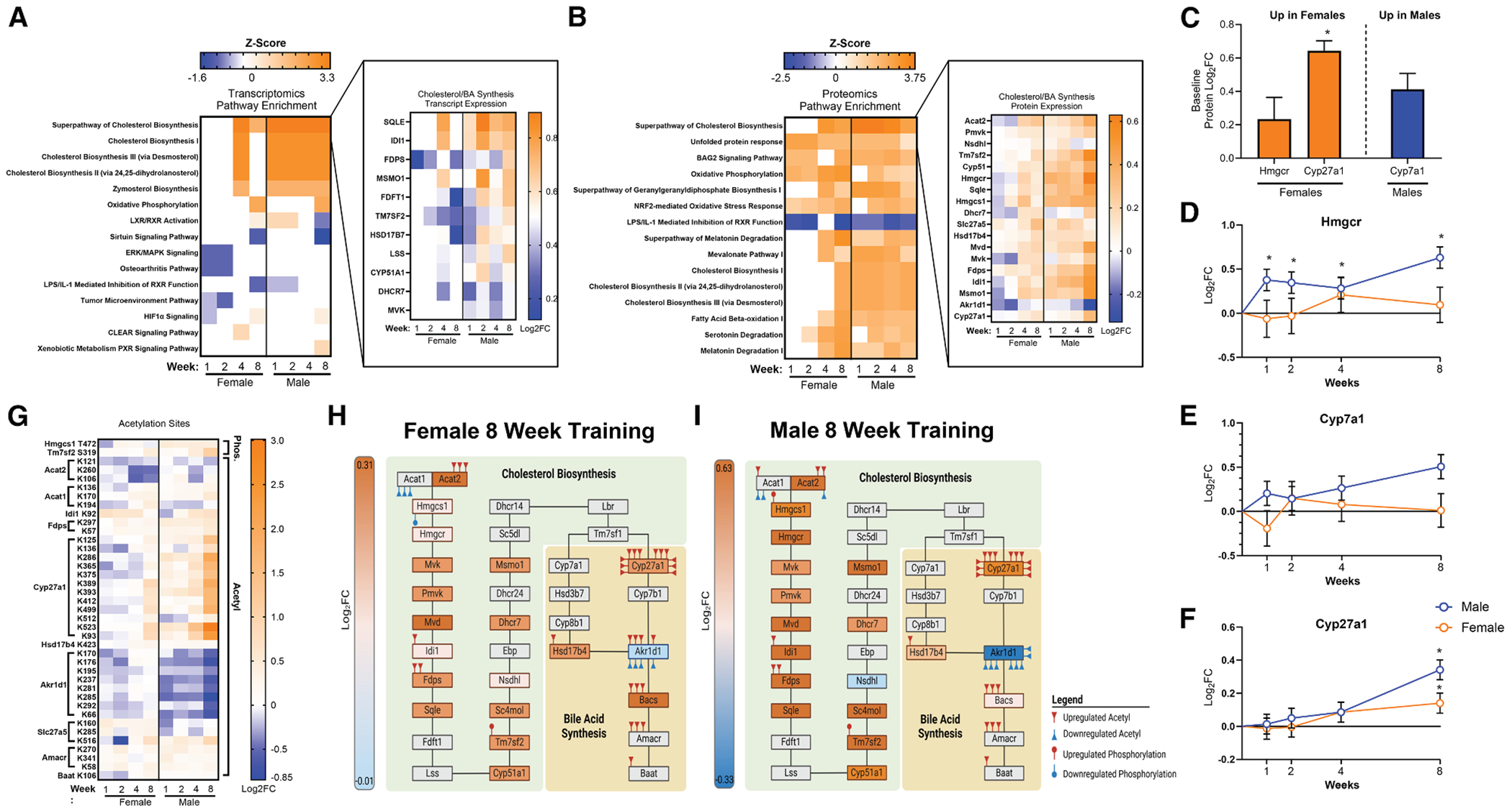
Liver cholesterol and BA biosynthesis are persistently increased in males but temporally delayed in females with chronic exercise training. (A and B) Comparison analysis heatmaps of top regulated biological functions from IPA transcriptomics (A) and proteomics (B) analysis after 1, 2, 4, or 8 weeks of treadmill running with expression of genes associated with the super pathway of cholesterol biosynthesis. (C) Sex differences in the abundance of the major regulatory proteins of cholesterol and BA synthesis pathways in sedentary control rats. (D–F) Time-point-specific responses to treadmill training of HMGCR (D), CYP7A1 (E), and CYP27A1 (F) at 1, 2, 4, or 8 weeks of training. (G) Heatmap of cholesterol and BA synthesis and post-translational modification (PTMs) for phosphorylation and acetylation events after 1, 2, 4, or 8 weeks of treadmill running. (H and I) Protein and PTM visualization of cholesterol and BA synthesis pathway after 8 weeks of treadmill running in male (H) and female rats (I). DEPs with *Z* score > 2 were considered activated. Abundance levels of DAPs are displayed as log2FC. Phosphoproteome and acetylome values are expressed as log2FC. Blue and green colors represent a decrease and orange and red an increase in endurance training compared to sedentary rats (*n* = 5/sex/condition). Values are represented as mean ± standard error. (H) and (I) are generated in BioRender.

Endurance exercise training analyte results (transcript and protein; FDR < 0.05) were imputed into IPA to generate mechanistic networks for top-predicted biological functions and upstream regulators (absolute *Z* score > 2; Figures 6A and 6B). IPA revealed sex- and time-dependent activation of cholesterol biosynthesis with induction of cholesterol biosynthesis across all time points in the males but only at 4 and 8 weeks in females (absolute *Z* score > 2; Figures 6A and 6B).

We examined hepatic abundance of HMGCR, the rate-limiting enzyme in cholesterol synthesis,^56^ and CYP27A1, which initiates the alternative BA pathway.^57^ Both proteins were higher in sedentary females than males (Figure 6C). After 8 weeks of training, CYP27A1 increased in both sexes, whereas HMGCR increased only in males (Figure 6D). CYP7A1, the rate-limiting enzyme of the classical BA pathway,^58^ did not change significantly but followed trends similar to HMGCR (Figure 6E). CYP27A1 was also highly acetylated for 8 weeks (Figure 6G). Additional cholesterol/BA pathway proteins, including SLC27A5, which facilitates BA conjugation,^59^ and SCP-2, which stimulates CYP7A1 activity, were upregulated with exercise (Figure 2B). Integrated multi-omics summaries of cholesterol/ BA pathway protein and PTM changes at 8 weeks are shown for females and males in Figures 6H and 6I, respectively.

While both sexes increase cholesterol biosynthesis with distinct temporal patterns, further analysis revealed that cholesterol and BA pathways are elevated in female rats compared with males in sedentary conditions (absolute *Z* score > 2; Figures S3A–S3E). Upstream regulators of cholesterol and phosphatidylcholine synthesis in the liver of males and females after 8 weeks of treadmill running in females were also observed at the transcript and protein level (absolute *Z* score > 2; Figures S4A–S4D).

### Chronic exercise training reduces liver lipids and changes fecal excretion of lipids in a sexual dimorphic manner

**Figure 7.**
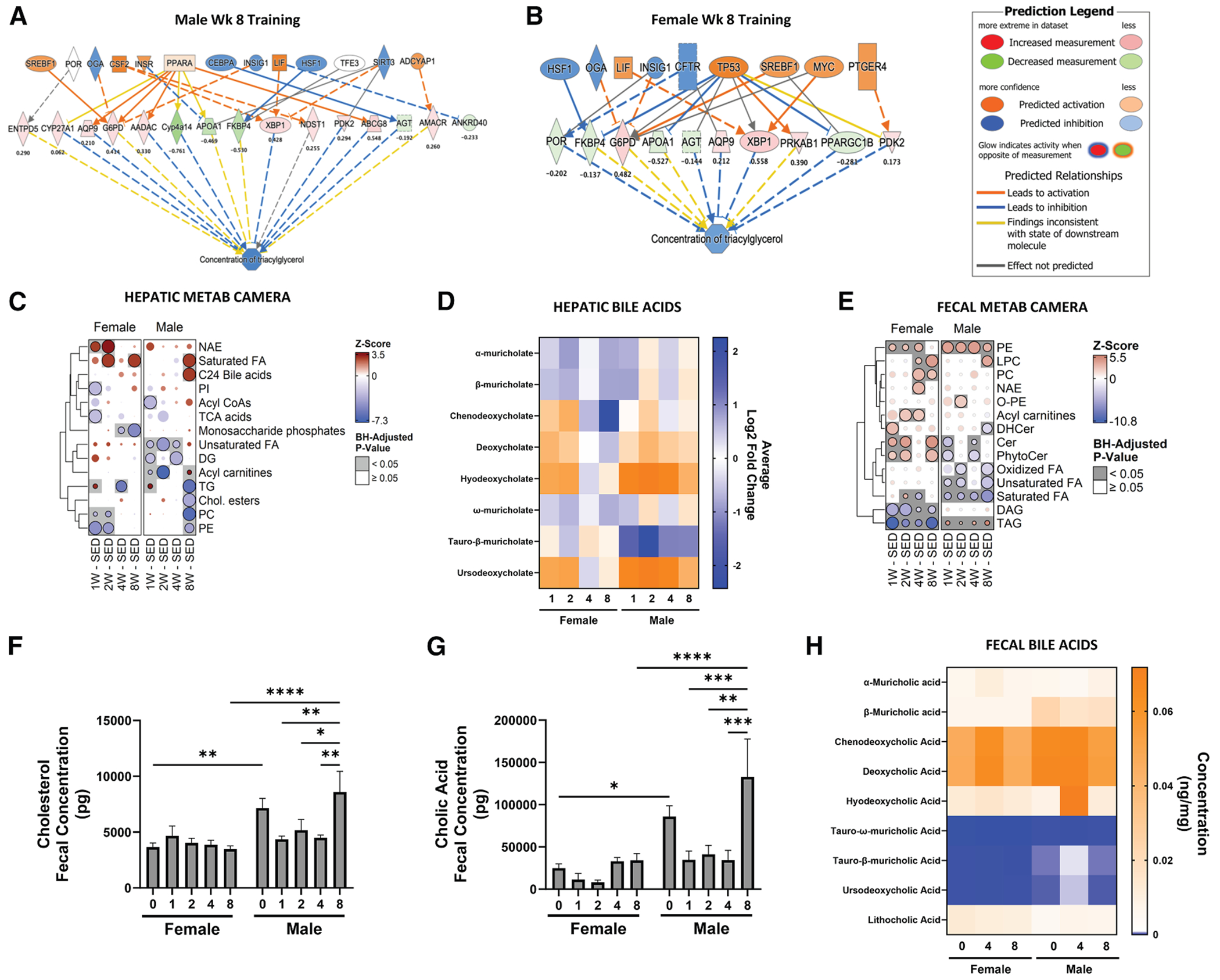
Chronic exercise training reduces lipids in the liver and changes fecal excretion of lipids in a sexual dimorphic manner. (A and B) Transcriptomic-activated upstream regulators predicted by IPA display a sex-consistent decrease in triacylglycerol concentration after 8 weeks of treadmill running in male (A) and female (B) rats. Blue and green colors represent a decrease, and orange and red represent an increase in endurance training compared to sedentary rats (*n* = 5). DEGs are shown below each node and displayed as log2 fold change. DEGs with *Z* score > 2 were considered activated. (C) Bubble heatmap depicting top biological functions (BF) from the Gene Ontology (GO) database across training. The heatmap was derived from the liver metabolic CAMERA-PR results methods. Circles are colored by the *Z* score and scaled by row so that the most significant comparison is of maximum area. (D) Heatmap showing liver BA concentration after training (1−, 2−, 4−, and 8-week SED) in male and female rats (*n* = 5). (E) Bubble heatmap depicting top biological processes (BP) from the GO database across training. The heatmap was derived from the fecal metabolic CAMERA-PR results. (F and G) Male and female fecal cholesterol (F) and cholic acid (G) concentration at sedentary levels and after 1, 2, 4, and 8 weeks of treadmill running (*n* = 5/sex/condition). (G) Fecal BA species and summated fecal BA concentration at sedentary levels and after 4 and 8 weeks of treadmill running in male and female rats (*n* = 7–8/sex/condition). A gray or black background indicates a significant result (adjusted *p* value < 0.05). Values are represented as mean ± standard error. *Indicated significance, *p < 0.05, **p < 0.01, ***p < 0.001, ****p < 0.0001.

Exercise has a profound effect in lowering intrahepatic lipid stores.^12,22–24,55,60–66^ Therefore, we also explored exercise-induced changes in pathways controlling triacylglycerol (TG) primary storage depots and intermediate pools. IPA predicted decreased concentrations of TG after 8 weeks of endurance training (Figures 7A and 7B). Metabolic analysis corroborated IPA transcriptomic predictions, revealing a significant decrease in liver TG, cholesterol esters (CE), phosphatidylcholines (PC), and phosphatidylethanolamines (PE) in the male livers after 8 weeks of training (*p* < 0.05; Figure 7C). Liver metabolomic enrichment also displayed a significant increase in C24 BAs in male livers after 8 weeks of exercise (*p* < 0.05; Figure 7C), with deoxycholate, hyodeoxycholate, and ursodeoxycholate comprising the majority of the BA pool (Figure 7D). Deoxycholate, hyodeoxycholate, and ursodeoxycholate were quantitatively higher (not significant) across all exercise time points for males but peaked at week 2 for females, followed by a gradual decrease. Interestingly, no significant changes in plasma lipid concentrations were detected in males. However, plasma concentration of cholesterol (one-way ANOVA, *p* = 0.0479), DEGs (one-way ANOVA, *p* = 0.001), and TGs (one-way ANOVA, *p* = 0.00260) were reduced with exercise in the females. Post hoc analyses revealed that cholesterol was reduced after the first week of training with DEGs, and TGs were reduced after 1, 2, and 4 weeks of training in females (*p* < 0.05; Table S1).

The predicted elevation of cholesterol and BA synthesis in the liver (Figure 7) led us to investigate if an increased fecal loss of lipids, BAs, and cholesterol was causing an upregulation of synthesis to maintain systemic BA and cholesterol homeostasis. Males and females had increases in PE and lysophosphatidylcholine (LPC) with exercise (*p* < 0.05; Figure 7E). Furthermore, exercise in females also increased fecal PC, N-acylethanolamine (NAE), acyl-carnitines, and ceramides (cer) (*p* < 0.05; Figure 7E). There was also a sexual dimorphic response to chronic exercise. Exercise training increased fecal TGs in males but reduced them in females (*p* < 0.05; Figure 7E). Typically, approximately 5% of the BA pool is lost in the stool, while 95% is recycled by the liver; however, rats bred for high aerobic capacity have an approximately 50% increase in fecal BA loss compared to rats bred for low aerobic capacity.^67,68^

Analyses of fecal cholesterol concentration revealed a significant interaction between sex and exercise for fecal cholesterol (two-way ANOVA, *p* = 0.0080) and cholic acid concentration (two-way ANOVA, *p* = 0.0420). Fecal cholesterol was significantly elevated after 8 weeks of exercise compared to 1, 2, and 4 weeks of exercise in males (*p* < 0.05; Figure 7F). Moreover, fecal cholic acid concentration, a primary component of the BA pool, mirrored fecal cholesterol with a significant elevation after 8 weeks of exercise compared to 1, 2, and 4 weeks of exercise in males (*p* < 0.05, Figure 7G). However, females did not display increased fecal cholesterol or cholic acid. Other pools of fecal BAs were not significantly changed with exercise in either sex (Figure 7H). These data provide evidence of an exercise-induced increase in liver BA synthesis that could mediate liver and fecal cholesterol/lipid excretion in a sex-specific manner.

## DISCUSSION

Previous reports demonstrate that female rodents possess elevated liver mitochondrial protein content^69,70^ and greater individual complex protein abundance,^13,34^ but these analyses often rely on the abundance of a single protein within OXPHOS complex proteins or bulk quantification of proteins. To our knowledge, MoTrPAC,^35^ was the first study to utilize proteomicsbased approaches to quantify changes in both mitochondrial protein abundance and mitochondrial acetylation events following successive weeks of endurance exercise training in male and female rats. Here, we averaged the protein abundance of OXPHOS to provide a comprehensive readout. With exercise, females increased OXPHOS protein abundance across all complexes except V to a greater extent than males, exceeding overall mitochondrial protein gains, indicating preferential OXPHOS expansion. These adaptations may involve estrogen-mediated regulation of mitochondrial biogenesis,^71^ though transient signaling may explain the absence of detectable biogenesis markers 48 h post-exercise.

The combined enrichment of metabolic processes and OXPHOS demonstrates a unique, paired increase in the rate of substrate catabolism and OXPHOS complex abundance induced by endurance training in female rats. Collectively, these mitochondrial alterations may support enhanced gluconeogenic capacity during exercise, allowing female rodents to maintain a larger volume of exercise,^72^ as implicated by expressional changes in PFKL, ENO1, PCK2, and PDK4. These findings support the previous findings in female mice, including more robust changes in mitochondrial respiratory capacity to metabolic stress,^13^ greater mitochondrial coupling control, and an enhanced pairing of oxygen consumption to ATP production in female livers compared to males.^13,32^

Sex differences in the TCA cycle and OXPHOS protein abundance suggest distinct metabolic handling between males and females. Females likely couple TCA flux tightly to mitochondrial respiration to support energy production, consistent with greater respiratory capacity,^13,34^ whereas males may preferentially divert TCA intermediates toward cataplerotic and anabolic pathways independent of increased OXPHOS abundance. This decoupling may explain the limited OXPHOS remodeling in males despite elevated baseline metabolic protein levels and is supported by evidence of enhanced TCA cataplerosis and anaplerosis in male livers independent of respiration.^73^

Males also exhibited a robust increase in acetylation of mitochondrial proteins following exercise training. Despite limited expressional changes of the majority of acetyltransferase and deacetylase enzymes, we observed a robust elevation of CrAT in male rats across all training time points that was not observed in the females. Classically, CrAT mediates export of acetyl groups from the mitochondrial matrix by converting acetyl-CoA to acetyl carnitine, regulating the levels of free CoA pools. However, evidence in skeletal muscle suggests that exercise can reverse normal CrAT-mediated acetyl carnitine flux, preferentially promoting increased acetyl-CoA levels.^74^ Due to the bidirectional activity of CrAT, we hypothesized that CrAT acetylation in males may serve as a mechanism by which male rats preferentially accumulate acetyl-groups within the mitochondria, exceeding the capacity of mitochondrial deacetylases^40^ and increasing mitochondrial acetylation via mass action. Consistent with CrAT restoring acetyl-CoA concentrations reduced with exercise, hepatic acetyl-CoA levels were significantly decreased in the first couple of weeks of exercise training. Although acetyl-CoA was reduced in both males and females, only males showed increased mitochondrial protein acetylation, which we speculate is due to the innately high levels of CrAT in females.

CrAT overexpression in primary hepatocytes increased aggregated mitochondrial acetylation under high-glucose conditions, which was reduced under low-glucose conditions, confirming that CrAT can act bidirectionally depending on substrate/energy abundance. CrAT overexpression under high glucose conditions resulted in several individual mitochondrial proteins’ acetylation events in the TCA cycle, FAO, and OXPHOS pathways that were consistent with exercise training in males. The more robust increases in mitochondrial acetylation were observed under high glucose conditions, suggesting that higher levels of glucose are required for CrAT-induced acetylation. Taken together, these studies support the notion that exercise training results in conditions similar to those of high glucose exposure, reversing the activity of CrAT and increasing mitochondrial acetylation.

Training robustly increased cholesterol biosynthesis-related analytes across multiple omes in males, whereas this response was delayed in females. This delay likely reflects higher baseline mitochondrial function and cholesterol pathway activity in female livers. Females exhibit greater hepatic mitochondrial respiratory capacity and an enhanced ability to expand respiration under metabolic stress independent of oxidative stress,^13,32,34^ potentially reducing the acute impact of training. Similar sex differences may underlie delayed activation of cholesterol and BA synthesis pathways, which are elevated in females in part through estrogen-mediated signaling (82).

Markers of cholesterol and BA synthesis were also robustly increased following chronic exercise training. Proteomics revealed upregulation of HMGCR, the rate-limiting enzyme in cholesterol biosynthesis,^56^ by 4 weeks in both sexes, and increased abundance and acetylation of CYP27A1, the rate-limiting enzyme in the alternative BA pathway,^57^ by 8 weeks. Exercise also increased SLC27A5, which facilitates BA conjugation,^59^ and SCP-2, a regulator of CYP7A1 activity.^75^ Metabolomics further demonstrated evidence of increased hepatic BA synthesis after 8 weeks of exercise in males. Together, these data indicate exercise-induced activation of cholesterol and BA synthesis in both sexes, with delayed responses in females compared to males. In males, increased BA synthesis coincided with reduced hepatic lipid species (TG, CE, PE, PC), elevated fecal lipids, and increased cholic acid, a primary BA involved in cholesterol excretion (84). Because fecal BA secretion is a primary route of cholesterol elimination,^76^ we posit that enhanced cholesterol and BA synthesis may contribute to exercise-induced reductions in hepatic cholesterol.

These data are consistent with our previous findings that exercise training combined with diet-induced weight loss increased markers of liver BA synthesis^77^ and that high aerobic capacity and exercise are associated with upregulated BA synthesis and greater fecal excretion of cholesterol/BA in rats fed a high-fat diet.^67^ Furthermore, BA sequestrants have been shown to reduce liver lipids in mice,^78,79^ suggesting that exercise-mediated fecal BA loss may have a significant role in the treatment of steatosis. These data provide additional support for our recent findings that exercise-induced increases in BA synthesis and fecal BA loss are critical for exercise to reverse dietary-induced hepatic steatosis.^80^

Despite increased cholesterol and BA synthesis in both males and females, fecal cholesterol and cholic acid were not significantly altered by exercise in female rats. Given that these rats were on a standard low-fat chow diet, we speculate that females simply have less excess cholesterol available for elimination due to lower hepatic cholesterol content. Based on our previous findings,^11^ we would expect to observe a similar excretion of cholesterol with endurance training in conditions where females have higher hepatic lipid content (i.e., models of diet-induced obesity). More importantly, our recent work has shown that exercise-induced increases in hepatic BA synthesis and fecal excretion are obligatory for exercise to reverse high-fat diet-induced hepatic steatosis in both male and female mice, findings that were leveraged using inducible liver-specific *Cyp7a1* knockout mice. These reports suggest that the upregulation of global cholesterol biosynthesis, a substrate for BA metabolism, has significant health ramifications related to MASLD and possibly also in relation to systemic cholesterol homeostasis, which also impacts cardiovascular disease.

Decreased proliferation of HSCs/fibrosis was identified as a cell-type-specific adaptation with exercise. These included *AGT* and *CCND1*. AGT is converted to angiotensin in the liver,^81^ which promotes HSC activation and fibrosis through profibrotic pathways such as TGF-β1 signaling and oxidative stress.^49,50^ Overactivation of the renin-angiotensin system (RAS) is a known driver of fibrogenesis that exercise protects against,^82^ potentially through downregulation of AGT. CCND1 regulates cycle progression from G1 to S phase and is associated with proliferation and activation of HSCs.^51^ Exercise can revitalize quiescent skeletal muscle stem cells through cyclin D1,^83^ but this is the first study to report that chronic exercise decreases CCND1 protein abundance in the liver.

The combination of multi-omics approaches (transcriptome, proteome, phosphoproteome, acetylome, metabolome, and lipidome), time course, and inclusion of both biological sexes in this dataset provides an unprecedented perspective on the liver adaptations to endurance exercise training. Moreover, the results point to a marked sexual dimorphism in liver metabolism and mitochondrial profiles both in sedentary conditions and in temporal responses to endurance exercise training. Several exercise-induced adaptations only occur after longer durations of training, suggesting that for hepatoprotective effects, long-term endurance is likely most efficacious. The multi-omics data mining tools found at the bioinformatics core (MoTrPAC BIC; https://motrpac-data.org) can be leveraged for hypothesis-driven mechanistic studies to further understand the effects of exercise training on the liver, particularly at the mitochondrial level. Additionally, the robust exercise-induced changes in OXPHOS, cholesterol/BA synthesis, and mitochondrial acetylation point to novel targets to potentially combat metabolic diseases centered in the liver, including MASLD.

### Limitations of the study

These studies provided valuable insights into liver adaptations in response to exercise across 1, 2, 4, and 8 weeks of exercise, which would be unattainable in human participants. While rat models do align with several observations from humans,^38^ they do not fully replicate findings in humans.^84^ Differences in the metabolic rate,^85^ body-to-surface area, the lack of a gallbladder, muscle fiber composition,^86^ and other unknown factors that differ between rats and humans likely influence molecular adaptations to exercise. Additionally, while these studies comprehensively assessed molecular markers of exercise adaptation, this study did not include functional measurements such as mitochondrial respiration or metabolic flux. Nor did these studies incorporate DNA methylation patterns that could provide greater insight into the origins of sex differences within the liver. Further-more, these studies provided data 48 h post-training, missing transient responses that are not due to chronic training per se but are induced by each bout of exercise. Finally, all of the omes were measured in bulk liver tissue, including both parenchymal and non-parenchymal cell types. Future studies should examine the effects of endurance exercise on all 4 cell types in the liver with newer technologies (i.e., single-cell RNA sequencing, special metabolomics, etc.).

## RESOURCE AVAILABILITY

### Lead contact

- Requests for further information and resources should be directed to the lead contact, R. Scott Rector (rectors@health.missouri.edu).

### Materials availability

- This study did not generate new, unique reagents.

## STAR★METHODS

### EXPERIMENTAL MODEL AND STUDY PARTICIPANT DETAILS

#### Animals

Adult male and female Fischer 344 inbred rats were acquired from the National Institute of Aging (NIA) rodent colony. Upon arrival, rats were adapted to a 12-h reverse light-cycle, ensuring treadmill training occurred during the active period. Rats were housed as same-sex pairs in ventilated racks (Thoren Maxi-Miser IVC Caging System) on Tekland 7093 shredded Aspen bedding and fed *ad libitum* with Charles River Rat and Mouse 18% (Auto) 5L79 LabDiet (Gateway Lab Supply, St. Louis, Missouri), which has the following calorie composition: 21.196% protein, 14.774% fat (ether extract), 64.030% carbohydrates. These are the standard bedding and diet used at the NIA rodent colony. The animal housing room was monitored daily and was maintained at a temperature of 20°C–25°C and relative humidity of 25–55%. Red lights were used during the dark cycle to provide adequate lighting for routine housing tasks, rodent handling, and training. All animal procedures were approved by the Institutional Animal Care and Use Committee at the University of Iowa (Animal protocol #7082041-006), where the rats were housed. Complete description of animal husbandry described previously.^37^

#### Primary hepatocyte Isolation and adv-CrAT overexpression

A cohort of C57BL/6J mice (no. 000664, Jackson Laboratory) fed a standard chow (Formulab 5008, Purina Mills) was used for primary cell studies. Primary hepatocytes were isolated from 15-week-old male mice using a 2-step collagenase method described previously.^87^ Cellular preparations were assessed for viability using trypan blue and counted using hemocytometry. Preparations with >90% viability were used. Isolated hepatocytes were then plated on collagen-coated plates for 72 h. CrAT overexpression occurred immediately upon plating. To overexpress CrAT, primary hepatocytes were transfected with adenovirus (adv;1e6 viral particles) expressing mouse full-length CrAT (Adv-CrAT; Vector Biolabs) or enhanced green fluroscent protein control (Adv-eGFP); Vector Biolab) for 6 h as previously reported.^88^ Primary hepatocytes were exposed to moderate (11 mM) or high glucose (25mM) on day 2 (24 h after plating). On Day 3 cells were lysed in protein lysis buffer and collected for global and acetylome enriched proteomics. RNA was extracted from primary hepatocytes using an RNeasy Mini Kit (no. 74104; QIAGEN) and reverse transcribed (Promega) to create cDNA. Quantitative real-time PCR (qRT-PCR) was conducted using SYBR Green reagents (172–5121; Bio-Rad Laboratories, Inc) and primer pairs for CrAT Forward: 5′-3′ACAGCCTATCTCCAGTTCCG; Reverse: 5′-3′AATCCAATACACCCTCGATGAG) and PPIB (Forward: 5′-3′ TGGAGATGAATCTGTAGGAC; Reverse: 5′-3′ CAAATCCTTTCTCTCCTGTAG). Data are represented relative to cycoliphillin B (PPIB) using the 2-ΔΔCT method.

### METHOD DETAILS

Treadmill familiarization and training was conducted on a Panlab 5-lane rat treadmill (Model LE8710RTS, Harvard Instruments). All animal handling and exercise sessions were performed in the active, dark phase of the rats. Following acclimation, rats underwent familiarization for 12 days to acclimate rats to the treadmill and identify potential non-compliant rats. Rats that were unable to run on the treadmill for 5 min at a speed of 10 m/min and grade of 0° were classified as non-compliant and removed from the study. Rats that successfully completed the 12-day familiarization protocol were randomly assigned to a training or control group. Training began at 6 months of age and lasted 1,2,4, or 8 weeks. Rats were exercised for 5 consecutive days per week using a progressive protocol designed to maintain an intensity of 70% of VO_2_ max (increasing grade and speed, see MoTrPAC^35^ for details) reaching 50min/day at a 10° grade. To control for any non-exercise-related treadmill effects, sedentary control rats were placed on the treadmills for 15 min/day at a speed of 0 m/min for 5 consecutive days per week, following a schedule similar to the 8-week-trained rats. Rats unable to exercise for at least 4 days per week were removed from the study.

#### Tissue collection

Tissues were collected 48 h following the last exercise bout. On the day of collection, food was removed at 8:30 a.m., 3 h prior to the start of dissections, which occurred between 11:30 a.m. and 2:30 p.m. (in the dark cycle). Rats were anesthetized with 1–2% isoflurane, blood was drawn via cardiac puncture, the liver and fecal (from the rectum) were removed and immediately flash-frozen in liquid nitrogen and stored at −80°C. Cardiac exsanguination resulted in death. Rat tissues were archived at the MoTrPAC Biospecimens Repository, until distributed to Chemical Analysis Sites for respective assays. For a more detailed description please see associated MoTrPAC publication.^35^

### QUANTIFICATION AND STATISTICAL ANALYSIS

#### Multi-omic data generation and processing

For complete detailed descriptions of methods used for sample preparation and multi-omics data generation and processing at chemical analysis sites for this study (transcriptomics, proteomics, phosphoproteomics and metabolomics/lipidomics), please see the associated MoTrPAC publication.^35^ Sample preparation and multi-omics data generation and processing were performed the same as the associated MoTrPAC publication^35^ except where specified. At all multi-omic analysis sites, an unblinded batching officer managed the randomization of samples across appropriately sized batches for available analysis platforms. These randomized samples were blinded to all individuals involved in sample preparation, data generation, and initial data processing. Downstream quality control and data analysis were conducted with knowledge of experimental conditions.

#### Untargeted lipidomics

Untargeted lipidomic analysis were performed as previously described.^35^ We performed semi-absolute quantification of the annotated lipids. The concentrations of each lipid species were calculated by normalizing their peak area by that of the internal standard (ISTD) from its own class and the same ESI mode and then multiplying by the known ISTD concentration. The closest match was selected when a ISTD was not detected or if no ISTD available. After calculating the relative lipid abundance, we calculated the lipid subclass sums for each sample and identified outliers on the lipid subclass level within each ESI mode. Sample-level lipid sub-classes that were more than 5 median absolute deviations from the median were identified as outliers, and the entire lipid class was removed. The missing values were imputed using the NIPALS algorithm from the mixOmics package.^89^ The resulting negative imputed values for 19 TG species were replaced by feature mean. ISTD-normalized data are reported as μg/mg tissue for liver, μg/mg for plasma, or pg for fecal lipidomics.

#### Fecal lipidomics

Fecal samples (100 mg) were first homogenized with 1 mL homogenizer solution consisting of a 1:4 volumetric ratio of water:methanol. Samples were then centrifuged at 10000g for 5 min at 4°C. The supernatant was then plated. Using the Biotage Extrahera, a methanol crash was performed with Solid Phase Extraction. 1500 μL of Methanol was added to the samples prior to eluting the samples through a methanol preconditioned filter. The collected eluant was then dried with nitrogen gas and constituted in 200 μL of 1:1 volumetric ratio of Acetonitrile:Methanol and stored at −80°C until analysis. Chromatography was run on Agilent 6495C with 1290 Infinity II on Agilent Zorbax Eclipse Plus C18 (2.1 × 100 mm, 1.8 μm) column at flow rate of 0.5 mL/min at 65°C during a 21 min gradient. The mobile phase of UPLC grade solvents consisted of solvent A: 0.1% formic acid in water and Solvent B: 0.1% formic acid in acetonitrile. The injection volume of sample was 5 μL. Analysis was completed in negative mode. The Nozzle Voltage at −2000 V, Sheath Gas Flow at 11 L/min and Sheath Gas Temp at 400°C. Elution gradients and transition list are provided below. Sample data was calibrated against an external standard solution that was used to optimize instrumental parameters and establish limits of detection. Peak determination, peak area integration, and calculation of calibration curves for standards was performed with the Agilent MassHunter Workstation version 10.1.

#### CrAT global and acetylation enriched proteomics

Mouse hepatocyte lysate samples were reduced by incubation with TCEP (5 mM final) at 55°C for 30 min. Cysteine residues were alkylated by the addition of iodoacetamine (IAA, 10 mM final) and incubated at room temperature for 30 min in the dark. Reduced and alkylated proteins were precipitated by the addition of 5 volumes of ice-cold acetone and were incubated at −20°C overnight. Samples were centrifuged at 14, 000 x g for 10 min at 4°C to pellet the proteins. The supernatant was removed and the pellet allowed to dry on the benchtop for 15 min. The pellets were resuspended in 50 mM TEAB pH 8.5, 5% SDS and the proteins were processed using the S-trap mini following the manufacturer protocol.^90^ Peptides were dried in the vacuum centrifuge and stored at −20°C until acetyllysine enrichment.

Digested peptides were enriched for acetylated lysine residues using PTM-104 (PTM Bio, Chicago, IL) following the manufacturer protocol. Briefly, 10 μL of PBS-washed beads were added to each peptide sample and incubated at 4°C overnight with gentle end-to-end rotation. The beads were pelleted at 1000 x g for 1 min at 4°C and the supernatant removed and saved. The acetyllysine peptide conjugated beads were washed a total of 3 times each with 200 μL of wash buffer I (100 mM NaCl, 1 mM EDTA, 20 mM Tris-HCl, 0.5% NP-40), wash buffer II (100 mMM NaCl, 1 mM EDTA, 20 mM Tris-HC) and LC-MS H_2_0. Beads were pelleted, and supernatant removed between washes. Conjugated peptides were eluted with 0.1% TFA, peptides dried in the vacuum centrifuge and stored at −20°C until analysis. The supernatants were combined with the reserved flow through, dried in the vacuum centrifuge, and stored in the −20°C until global analysis.

Peptides were resuspended in 0.1% formic acid (FA) and quantitated on a Nanodrop spectrophotometer at 205 nm (Thermo) prior to LC-MS/MS analysis. 1 μg of each sample was injected using the Vanquish *Neo* (Thermo) nano-UPLC onto a C18 trap column (PepMap *Neo* Trap, 0.3 mm × 5 mm, 5 μm particle size (Thermo)) using pressure loading. Peptides were eluted onto the separation column (Aurora Elite XT, 75 μm × 150 mm, 1.7 μm C18 particle size, ionopticks) heated to 40°C prior to ionization at the mass spectrometer. Briefly, peptides were loaded and washed for 5 min at a flow rate of 0.400 μL/min at 2% B (mobile phase A: 0.1% FA in water, mobile phase B: 80% ACN, 0.1% FA in water). Peptides were eluted over 100 min from 2 to 25% mobile phase B before ramping to 40% B in 20 min. The column was washed for 15 min at 100% B before re-equilibrating at 2% B for the next injection. The nano-LC was directly interfaced with the Orbitrap Ascend Tribrid mass spectrometer (Thermo) equipped with a high field asymmetric ion mobility source (FAIMS).^91^ The data were collected by data dependent acquisition with the intact peptide detected in the Orbitrap at 120,000 resolving power from 375 to 1500 m/z. Peptides with charge +2–7 were selected for fragmentation by higher energy collision dissociation (HCD) at 28% NCE and were detected in the ion trap at rapid scan rate for the global analysis and in the Orbitrap at 30,000 resolving power for the acetyllysine enriched fractions. Dynamic exclusion was set to 60s after one instance. The mass list was shared between the FAIMS compensation voltages. FAIMS voltages were set at −45 (1.4 s), −60 (1 s), −75 (0.6 s) CV for a total duty cycle time of 3s. Source ionization was set at +1700 V with the ion transfer tube temperate set at 305°C. Raw files were searched against the mouse protein database downloaded from Uniprot on 08-18-2025 and a common contaminants database using SEQUEST in Proteome Discoverer 3.0.^92^ Search parameters consisted of static modification of cysteine (carbamidomethyl, +57.0125 Da) and dynamic modifications on methionine (oxidation, +15.9949), lysine (acetylation, +42.0106 Da), and protein N terminus (acetylation, +42.0106 Da) with a maximum of 3 modifications allowed per peptide. Abundances, abundance ratios, and *p*-values were exported to Microsoft Excel for further analysis. Avergae log2fc of mitochondrial proteins was used for aggragated mitochondrial protein acetylation. Statstitical signifiance was determined by a paired *t* test between high and low glucose groups. Standard error of the mean for CrAT protein expression was calculated based on Coefficient of Variation and aggregated mitochondrial protein acetylation based on individual acetylation events.

#### Analysis of training-responsive features

To identify training responsive features at any timepoint, male- and female-specific *p*-values were combined using Fisher’s sum of logs meta-analysis (F-test). These *p*-values were adjusted across all datasets within each ome to control the FDR using independent hypothesis weighting as in previous MoTrPAC publications.^35^ Training-differential features were selected at 5% FDR. Covariates were selected from assay-specific technical metrics that explained variance in the data and were not correlated with exercise training (i.e., RNA integrity number (RIN), median 5′-3′ bias, percent of reads mapping to globin, and percent of PCR duplicates as quantified with Unique Molecular Identifiers).

#### Differential analysis of temporally regulated features

Differential analysis of the multi-omics datasets was performed as described by MoTrPAC.^35,43^ Since sample variability appeared to be influenced by sex, with higher variability in female samples, male and female datasets were subsequently analyzed separately. Here, each training time point was compared against their sex-matched sedentary controls, and males were compared to females at the sedentary and 8-week-trained timepoints.

For RNA sequencing, filtered raw counts were input in accordance with DESeq2 workflow to obtain DEG’s.^93^ For proteomics, limma with empirical Bayes variance shrinkage was used to obtain DAPs.^94^ Specifically, likelihood ratio tests (DESeq2nbinomLRT, lrtest) or F-tests (limma) were used to compare the model with ome-specific technical covariates and training groups as a predictor variable (i.e., sedentary control, 1 week, 2 weeks, 4 weeks, 8 weeks) against a reduced model with only technical covariates. All metabolomics datasets were log_2_-transformed and analytes with >20% missing values were removed. Median sample–sample correlation was used to identify outlier samples, which were manually reviewed by the metabolomics sites. Due to the overlap in the coverage of different metabolomics and lipidomics assays, some metabolites/lipids were measured in multiple platforms. Redundant metabolites/lipids were curated by taking into consideration their properties (polarity, solubility, etc.) and their respective assay methodologies (extraction solvent, elution solvent, column, etc.). Specific curation steps for overlapping coverage are described in detail elsewhere.^95^

#### Correlation adjusted MEan RAnk gene set testing

The differential analysis results were collapsed to the gene (proteomics and transcriptomics), single phosphorylation site (phosphoproteomics), or Reference Set of Metabolite Names (RefMet) level by selecting the most extreme *Z* score for each combination of target feature and contrast. If any source features did not map to a target feature,^96^ the source feature ID was used to avoid unnecessarily removing data. These z-scores, along with Gene Ontology Biological Process gene sets,^45,46^ from the Molecular Signatures Database (MSigDB),^47,48^ phosphorylation sites grouped according to their known kinases from PhosphoSitePlus,^97^ and RefMet IDs grouped according to their RefMet chemical subclasses were used as input for CAMERA-PR. CAMERA-PR is a modification of the two-sample *t* test that accounts for inter-molecular correlation to more correctly control the false positive rate.^44^ CAMERA-PR was performed with the cameraPR.matrix function from the TMSig R/Bioconductor package (https://doi.org/10.18129/B9.bioc.TMSig).^98,99^

#### Graphical clustering of differential analysis results

For longitudinal pattern discovery, we used the MoTrPAC graphical clustering framework to model timewise differential abundance across sex and training duration similar to previous MoTrPAC publications.^35^ Differential analysis was performed at each training time point separately for males and females, generating *Z* score for each analyte. These timewise z-scores were model separated null, up-, and downregulated states. An expectation maximization approach was used to estimate probabilities for all possible sex- and time-dependent configurations. Identified differential analytes based each timepoint and transitions between states across adjacent time points were represented by a graphical trajectory. This approach accounts for sex-specific correlations while also avoiding limitations from standard clustering approaches.

#### Mitochondrial proteome identification

MitoCarta3.0 was referenced to isolate all mitochondrial proteins and the pathways they associate with from the entire liver proteome identified by the untargeted and acetyl-proteomics analysis, as previously described.^30,100^ To determine overall directional abundance of a given pathway or protein complex, the log_2_ fold-changes of all proteins within a pathway were averaged. This analysis was performed across-sex at sedentary levels, and within-sex for each training timepoint.

#### Ingenuity pathway analysis enrichment

Additional pathway and network analysis were performed using IPA software (QIAGEN Inc., Redwood City, California), similar to previous studies.^88,101–105^ Differentially expressed analytes (i.e., genes or proteins) and corresponding log_2_ fold changes were used as IPA input. IPA compared uploaded data to a curated knowledge base of biological interactions, pathways, and molecular relationships derived from peer-reviewed literature to identify enriched pathways and upstream regulators based on over-represented genes/protein clusters in the dataset. IPA was used to generate interactive visualizations (pathway diagrams, network, and heatmaps) and determines statistical significance based on *p*-value and z-scores of these pathways and upstream regulators. The canonical pathway tool was used to identify overrepresented pathways with a -log (*p*-value) threshold of 1.3 (Fischer’s Exact Test) and an absolute *Z* score >2 was used to determine pathway enrichment. Upstream Regulator analysis tool predicted transcription factors, kinases, and other regulatory molecules responsible for observed gene and protein abundances changes. Comparison analysis function in IPA was utilized to compare datasets from each timepoint and sex comparison to identify significant (absolute *Z* score>2) biological pathways. The sex-matched 8-week training differential features were further analyzed by identification of upstream regulators that were used to build disease and function pathways for the proteomic dataset. Canonical pathways and upstream regulators with a *Z* score exceeding ±2 generated by IPA’s proprietary prediction algorithm were considered significant (i.e., activated or inhibited).^106^

#### Statistical analysis software

The R programming language (A Language and Environment for Statistical Computing. R Foundation for Statistical Computing, Vienna, Austria. https://www.R-project.org) was used to perform statistical analyses and generate most figures with the remainder created with IPA (Qiagen Inc., Redwood City, California USA, https://digitalinsights.qiagen.com) or GraphPad Prism (GraphPad Software, Boston, Massachusetts USA, www.graphpad.com). Data and analysis tools for the MoTrPAC landscape paper^35^ are also provided through the MoTrPAC Training6moData and MoTrPAC Training6moData R packages, respectively (github.com/MoTrPAC/MotrpaCrATTraining6moData, github.com/MoTrPAC/MotrpacTraining6mo); the former package was used to access data for MoTrPAC Training6moData (github.com/PNNL-Comp-Mass-Spec/MotrpacTraining6moWATData). Statistical analyses for fecal cholesterol and cholic acid concentration were performed with GraphPad Prism version 10.1.2 (Prism) with α = 0.05. Two-way analysis of variance (ANOVA) was used with a Tukey test for multiple comparison procedure. One-way ANOVA was used for plasma lipids compared to sedentary (F0 or M0) state, respectively, with a Dunnett’s test for multiple comparison procedure. Bubble heatmaps were generated with the enrichmap function from the TMSig (10.18129/B9.bioc.TMSig) R/Bioconductor package.^98^ The value of ‘n’ represents Invidia animal number for *in vivo* experiments or a sample from an individual well for *in vitro* experiments. Value of ‘n’, precision methods, and other statistical details can be found in the figure legends. BioRender was used to generate Figures 3I and 5H–5I.

## Supplementary Material

1

2

3

4

Supplemental information can be found online at https://doi.org/10.1016/j.celrep.2026.117416.

## Figures and Tables

**Table:** KEY RESOURCES TABLE

REAGENT or RESOURCE	SOURCE	IDENTIFIER
Biological samples		
Fecal samples	MoTrPAC biorepository	
Critical commercial assays		
RNeasy Mini Kit	QIAGEN	no. 74104
Deposited data		
CrAT experiments mass spectrometry data	MassIVE/ProteomeXchange	MassIVE database: MSV000100983, MSV00101014 (global dataset). It has also been submitted to ProteomeXchange (database: PXD074952, PXD075090; acetyl enriched and global, respectively).
MoTrPAC data	https://motrpac-data.org/data-download	MoTrPAC omic datasets
Experimental models: Organisms/strains		
Fischer 344 rats	National Institute of Aging (NIA) rodent colony	
C57BL/6J mice	Jackson Laboratory	
Oligonucleotides		
Real-time PCR primers	Millipore Sigma	CrAT
Real-time PCR primers	Millipore Sigma	PPIB
Recombinant DNA		
Adenovirus	Vector Biolabs	Adv-eGFP
Adenovirus	Vector Biolabs	Adv-CrAT
Software and algorithms		
MoTrPAC data processing pipelines for RNA-Seq and proteomics	https://github.com/MoTrPAC/motrpac-rna-seq-pipeline and https://github.com/MoTrPAC/motrpac-proteomics-pipeline	
Code for the underlying differential analysis	MotrpacTraining6mo R package (motrpac.github.io/MotrpacTraining6mo)	

## Data Availability

MoTrPAC data are publicly available at https://motrpac-data.org/data-download. Data access inquiries should be sent to motrpac-helpdesk@lists.stanford.edu. Additional resources can be found at motrpac.org and motrpac-data.org. Primary hepatocyte CrAT experimential data can be found in repositories as described below. The mass spectrometry data have been deposited to MassIVE (https://massive.ucsd.edu/). The accession number for the acetyllysine-enriched data reported in this paper is MassIVE MSV000100983, and the global dataset is MSV000101014. It has also been submitted to ProteomeXchange (PXD074952 and PXD075090; acetyl-enriched and global, respectively). MoTrPAC data processing pipelines for RNA sequencing and proteomics is available at: https://github.com/MoTrPAC/motrpac-rna-seq-pipeline and https://github.com/MoTrPAC/motrpac-proteomics-pipeline. Normalization and quality control scripts will be available at https://github.com/MoTrPAC/motrpac-proteomics-pipeline/tree/master/scripts. Code for the underlying differential analysis for the manuscript will be provided in the MotrpacTraining6mo R package (motrpac.github.io/MotrpacTraining6mo). Any additional information required to reanalyze the data reported in this paper is available from the lead contact upon request.

## Highlights

- Male rats increased the acetylation of liver mitochondria proteins with endurance exercise
- Females increased the expression of liver mitochondria proteins with endurance exercise
- Chronic endurance exercise increased liver cholesterol and bile acid synthesis in both sexes
- Chronic endurance exercise increased lipid excretion in male rats

## CONSORTIA

MoTrPAC Study Group: Brent G. Albertson, Mary Anne S. Amper, Carter Asef, Matthew Babcock,Dam Bae, Marcas M Bamman, Bryan C. Bergman, Daniel H. Bessesen, Thomas W. Buford, Steven Carr, Chih-Yu Chen, Zachary S Clayton, Gary R. Cutter, Yongchao Ge, Laurie J. Goodyear, Kristy Guevara, Xueyun Gulbin, Michael F. Hirshman, Catherine M. Jankowski, Pierre M. Jean Beltran, Hasmik Keshishian, Wendy M. Kohrt, Sarah J. Lessard, Bridget Lester, Ana K. Lira, Nathan S. Makarewicz, Kristal M. Maner-Smith, Nada Marjanovic, Edward L. Melanson, Michael E. Miller, Samuel G. Moore, Kerrie L. Moreau, Charles C. Mundorff, Venugopalan D. Nair, German Nudelman, Nora-lovette Okwara, Paul D. Piehowski, Wei-Jun Qian, Blake B. Rasmussen, Stas Rirak, Irene E. Schauer, Nitish Seenarine, Cynthia L. Stowe, Yifei Sun, Scott Trappe, Todd A. Trappe, Mital Vasoya, Alexandria Vornholt, Michael P. Walkup, Ashley Xia, Xuechen Yu.
