# Supplementary material for: Endurance exercise elicits temporal and sexual dimorphic multi-omics remodeling of liver metabolism revealed by MoTrPAC
Source: Cell Rep. Author manuscript; Available in PMC 2026 Jul 20. (PMC13383671; doi:10.1016/j.celrep.2026.117416)
Supplement: 1 [file NIHMS2190714-supplement-1.pdf]

**Cell Reports, Volume *45***

## Supplemental information

# Endurance exercise elicits temporal and sexual dimorphic multi-omics remodeling of liver metabolism revealed by MoTrPAC

**Taylor J. Kelty, Edziu Franczak, Nicole R. Gay, Gina M. Many, Tyler J. Sagendorf, James A. Sanford, Zhenxin Hou, David A. Gaul, Facundo M. Fernández, Michaella Rekowski, Charles F. Burant, Andrea L. Hevener, Joshua N. Adkins, Sue Bodine, Malene E. Lindholm, Eric A. Ortlund, Simon Schenk, John P. Thyfault, R. Scott Rector, and the MoTrPAC study group**

| Symbol | Name |
|---|---|
| Acat1 | acetyl-CoA acetyltransferase |
| Acsm5 | acyl-CoA synthetase medium chain family member 5 |
| Adv | adenovirus |
| AGT | Angiotensinogen |
| ANOVA | Analysis Of Variance |
| Atp5bp | ATP synthase peripheral stalk-membrane subunit b |
| Atp5f1a | ATP synthase F1 subunit alpha, mitochondrial |
| BA | Bile Acid |
| Bckdha | Branched Chain Keto Acid Dehydrogenase E1 Subunit Alpha |
| Bckhdb | Branched Chain Keto Acid Dehydrogenase E1 Subunit Beta |
| BP | Biological Processes |
| CAMERA-PR | Pre-Ranked Correlation Adjusted Mean Rank Gene Set Testing |
| CCND1 | Cyclin D1 |
| CDC7 | Cell Division Cycle 7 |
| CE | Cholesterol Esters |
| Cer | Ceramides |
| CrAT | Carnitine Acetyltransferase |
| CYP27A1 | Sterol 27-Hydroxylase |
| DAPs | Differentially Abundant Proteins |
| DEGs | Differentially Expressed Genes |
| Derc1 | 2,4-dienoyl-CoA reductase 1 |
| Dld | dihydrolipoamide dehydrogenase |
| Eci1 | enoyl-CoA isomerase 1 |
| EGFP | enhanced green fluorescent protein |
| FDR | False Discovery Rate |
| GO | Gene Ontology |
| Hadha | Hydroxyacyl-Coa Dehydrogenase Trifunctional Multienzyme Complex Subunit Alpha |
| Hadhb | Hydroxyacyl-Coa Dehydrogenase Trifunctional Multienzyme Complex Subunit Beta |
| HMGCR | 3-Hydroxy-3-Methyl-Glutaryl-Coenzyme A Reductase |
| HSC | Hepatic Stellate Cell |
| IPA | Ingenuity Pathway Analysis |
| ISTD | Internal Standard |
| KSEA | Kinase–Substrate Enrichment Analysis |
| LPC | Lysophosphatidylcholine |
| MAP | Mitogen-Activated Protein |
| MASLD | Metabolic-Dysfunction-Associated Steatotic Liver Disease |
| Mcee | methylmalonyl-CoA epimerase |
| Mdh2 | malate dehydrogenase 2 |

| | |
|---|---|
| MoTrPAC | Molecular Transducers of Physical Activity Consortium |
| MSigDB | Molecular Signatures Database |
| mTOR | Mammalian Target of Rapamycin |
| NAE | N-Acylethanolamine |
| NIA | National Institute of Aging |
| OXPHOS | Oxidative Phosphorylation |
| PC | Phosphatidylcholines |
| Pdha1 | Pyruvate Dehydrogenase E1 Subunit Alpha 1 |
| Pdhb | Pyruvate Dehydrogenase E1 Subunit Beta |
| PE | Phosphatidylethanolamines |
| PTMs | Post-Translational Modifications |
| RAS | Renin-Angiotensin System |
| RefMet | Reference Set Of Metabolite Names |
| RIN | RNA Integrity Number |
| SCP-2 | Sterol Carrier Protein-2 |
| SLC27A5 | Solute Carrier Family 27 Member 5 |
| SREBP1c | Sterol Regulatory Element Binding Protein 1c |
| Sulcg1 | Succinate-Coa Ligase Gdp/Adp-Forming Subunit Alpha |
| Sulcg2 | Succinate-Coa Ligase Gdp/Adp-Forming Subunit Beta |
| TG | Triacylglycerol |
| TGF-β1 | Transforming Growth Factor Beta-1 |

**Data S1: Abbreviations.** Full name of abbreviations shown in the right column.

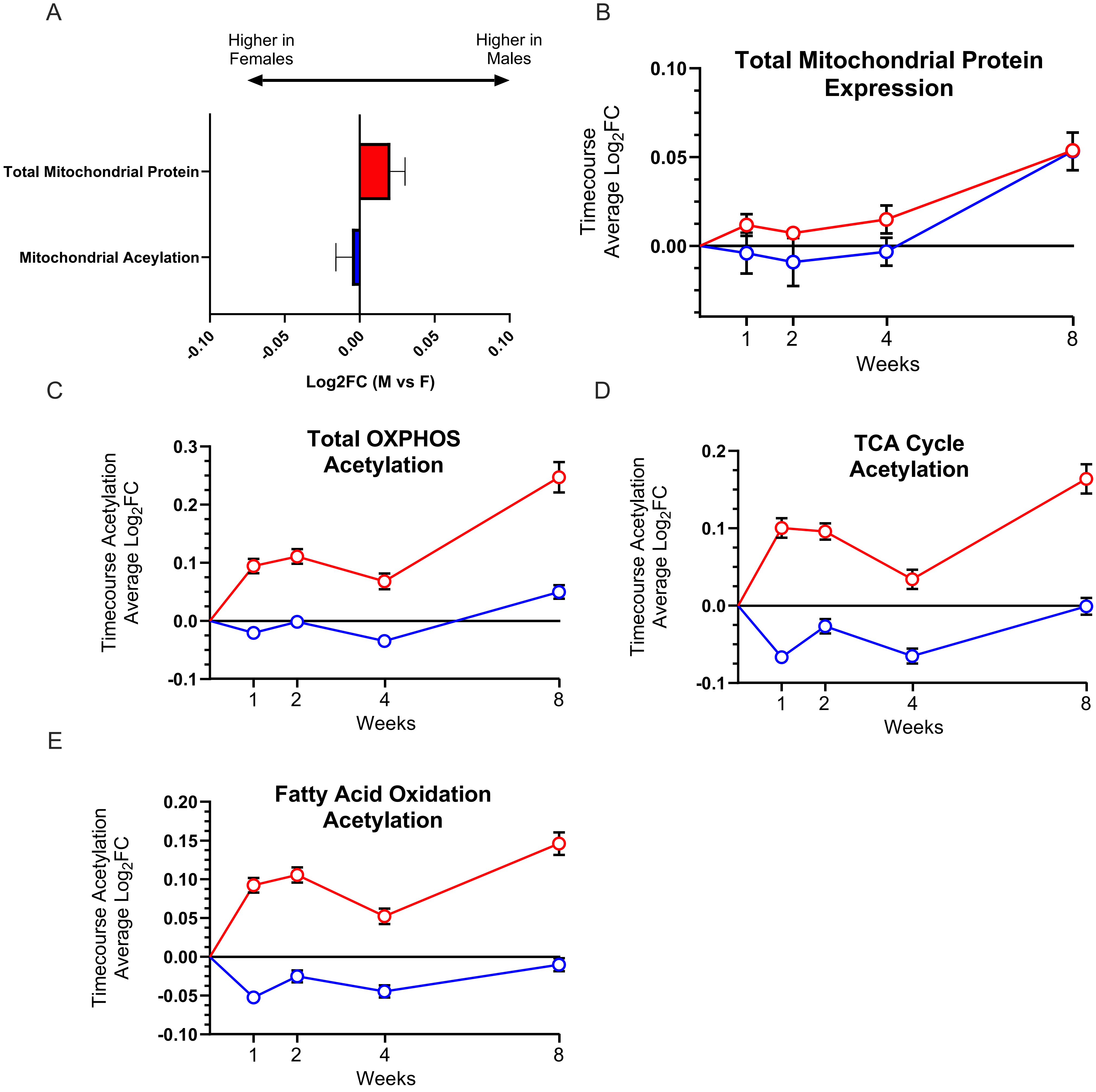
A
Higher in Females
Higher in Males
Total Mitochondrial Protein
Mitochondrial Aceylation
-0.10
-0.05
0.00
0.05
0.10
Log2FC (M vs F)
B
Total Mitochondrial Protein Expression
Timecourse Average Log2FC
0.10
0.05
0.00
1
2
4
8
Weeks
C
Total OXPHOS Acetylation
Timecourse Acetylation Average Log2FC
0.3
0.2
0.1
0.0
-0.1
1
2
4
8
Weeks
D
TCA Cycle Acetylation
Timecourse Acetylation Average Log2FC
0.2
0.1
0.0
-0.1
1
2
4
8
Weeks
E
Fatty Acid Oxidation Acetylation
Timecourse Acetylation Average Log2FC
0.20
0.15
0.10
0.05
0.00
-0.05
-0.10
1
2
4
8
Weeks

**Figure S1: Sedentary and exercise-mediated changes in the abundance of mitochondrial proteome and acetylome in the liver.** **A)** Average $log_2FC$ of total mitochondrial protein abundance and acetylation events between sedentary male and female rats. **B)** Exercise-mediated change in mitochondrial protein abundance presented as average $log_2FC$ for all mitochondrial protein. **C-E)** Pathway specific average $log_2FC$ in protein acetylation for OXPHOS, TCA cycle and fatty acid oxidation. **G)** CrAT abundance change over the exercise training paradigm. **H)** $Log_2FC$ of deacetylases and acetyltransferase enzymes between sedentary male and female rats. Values are represented as mean ± standard error.

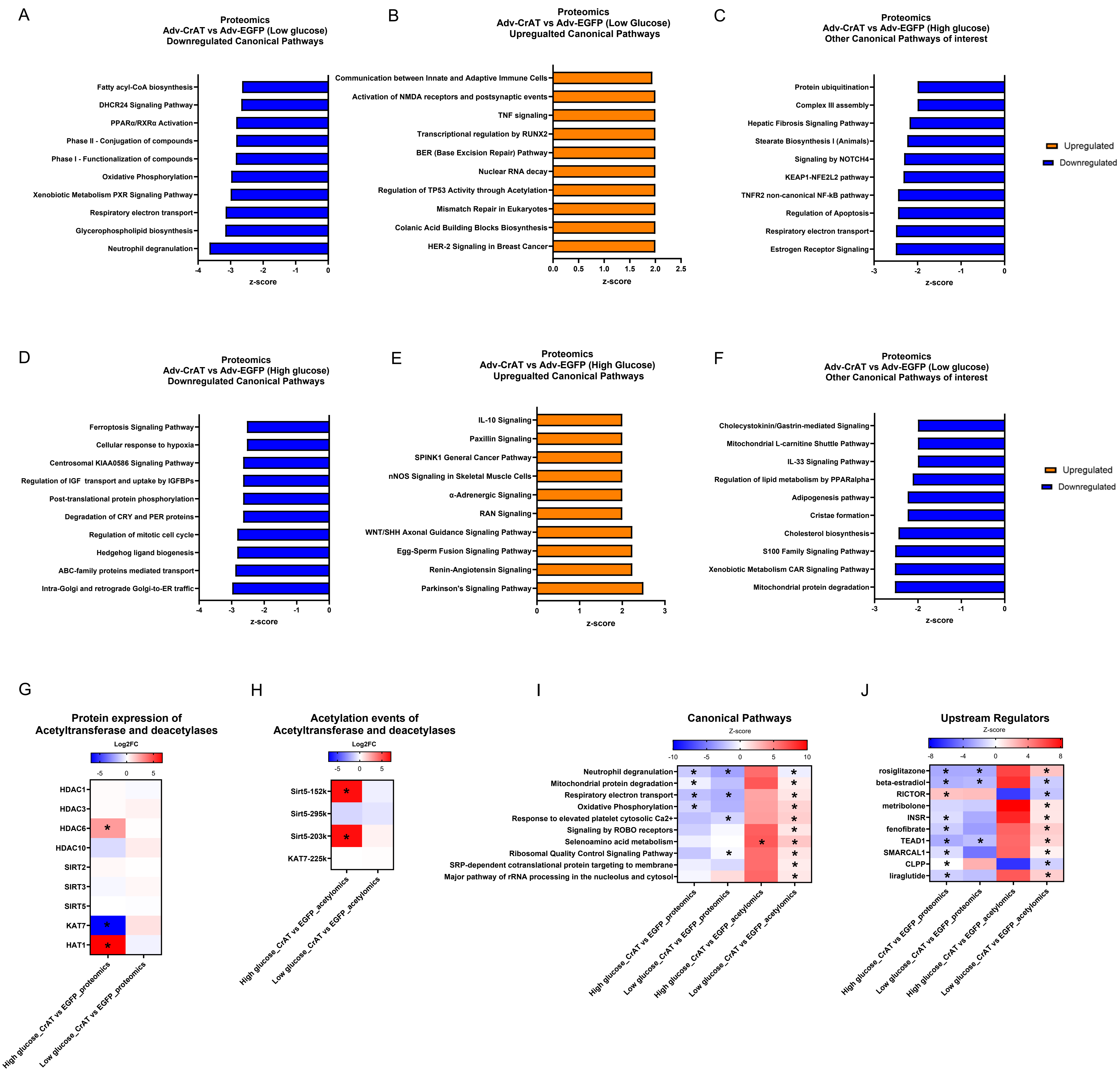
A
Proteomics
Adv-CrAT vs Adv-EGFP (Low glucose)
Downregulated Canonical Pathways
Fatty acyl-CoA biosynthesis
DHCR24 Signaling Pathway
PPARα/RXRα Activation
Phase II - Conjugation of compounds
Phase I - Functionalization of compounds
Oxidative Phosphorylation
Xenobiotic Metabolism PXR Signaling Pathway
Respiratory electron transport
Glycerophospholipid biosynthesis
Neutrophil degranulation
z-score
B
Proteomics
Adv-CrAT vs Adv-EGFP (Low Glucose)
Upregualted Canonical Pathways
Communication between Innate and Adaptive Immune Cells
Activation of NMDA receptors and postsynaptic events
TNF signaling
Transcriptional regulation by RUNX2
BER (Base Excision Repair) Pathway
Nuclear RNA decay
Regulation of TP53 Activity through Acetylation
Mismatch Repair in Eukaryotes
Colanic Acid Building Blocks Biosynthesis
HER-2 Signaling in Breast Cancer
z-score
C
Proteomics
Adv-CrAT vs Adv-EGFP (High glucose)
Other Canonical Pathways of interest
Protein ubiquitination
Complex III assembly
Hepatic Fibrosis Signaling Pathway
Stearate Biosynthesis I (Animals)
Signaling by NOTCH4
KEAP1-NFE2L2 pathway
TNFR2 non-canonical NF-kB pathway
Regulation of Apoptosis
Respiratory electron transport
Estrogen Receptor Signaling
z-score
Upregulated
Downregulated
D
Proteomics
Adv-CrAT vs Adv-EGFP (High glucose)
Downregulated Canonical Pathways
Ferroptosis Signaling Pathway
Cellular response to hypoxia
Centrosomal KIAA0586 Signaling Pathway
Regulation of IGF transport and uptake by IGFBPs
Post-translational protein phosphorylation
Degradation of CRY and PER proteins
Regulation of mitotic cell cycle
Hedgehog ligand biogenesis
ABC-family proteins mediated transport
Intra-Golgi and retrograde Golgi-to-ER traffic
z-score
E
Proteomics
Adv-CrAT vs Adv-EGFP (High Glucose)
Upregualted Canonical Pathways
IL-10 Signaling
Paxillin Signaling
SPINK1 General Cancer Pathway
nNOS Signaling in Skeletal Muscle Cells
α-Adrenergic Signaling
RAN Signaling
WNT/SHH Axonal Guidance Signaling Pathway
Egg-Sperm Fusion Signaling Pathway
Renin-Angiotensin Signaling
Parkinson's Signaling Pathway
z-score
F
Proteomics
Adv-CrAT vs Adv-EGFP (Low glucose)
Other Canonical Pathways of interest
Cholecystokinin/Gastrin-mediated Signaling
Mitochondrial L-carnitine Shuttle Pathway
IL-33 Signaling Pathway
Regulation of lipid metabolism by PPARalpha
Adipogenesis pathway
Cristae formation
Cholesterol biosynthesis
S100 Family Signaling Pathway
Xenobiotic Metabolism CAR Signaling Pathway
Mitochondrial protein degradation
z-score
Upregulated
Downregulated
G
Protein expression of
Acetyltransferase and deacetylases
Log2FC
HDAC1
HDAC3
HDAC6
HDAC10
SIRT2
SIRT3
SIRT5
KAT7
HAT1
High glucose_CrAT vs EGFP_proteomics
Low glucose_CrAT vs EGFP_proteomics
H
Acetylation events of
Acetyltransferase and deacetylases
Log2FC
Sirt5-152k
Sirt5-295k
Sirt5-203k
KAT7-225k
High glucose_CrAT vs EGFP_acetylomics
Low glucose_CrAT vs EGFP_acetylomics
I
Canonical Pathways
Z-score
Neutrophil degranulation
Mitochondrial protein degradation
Respiratory electron transport
Oxidative Phosphorylation
Response to elevated platelet cytosolic Ca2+
Signaling by ROBO receptors
Selenoamino acid metabolism
Ribosomal Quality Control Signaling Pathway
SRP-dependent cotranslational protein targeting to membrane
Major pathway of rRNA processing in the nucleolus and cytosol
High glucose_CrAT vs EGFP_proteomics
Low glucose_CrAT vs EGFP_proteomics
High glucose_CrAT vs EGFP_acetylomics
Low glucose_CrAT vs EGFP_acetylomics
J
Upstream Regulators
Z-score
rosiglitazone
beta-estradiol
RICTOR
metribolone
INSR
fenofibrate
TEAD1
SMARCAL1
CLPP
liraglutide
High glucose_CrAT vs EGFP_proteomics
Low glucose_CrAT vs EGFP_proteomics
High glucose_CrAT vs EGFP_acetylomics
Low glucose_CrAT vs EGFP_acetylomics

**Figure S2: Global proteomic and acetylomics pathways altered with CrAT overexpression in primary hepatocytes.**

**A-B)** Top 10 proteomic pathways **(A)** upregulated or **(B)** downregulated with CrAT overexpression under low glucose conditions. **(C)** Proteomic pathways of interest that were downregulated with CrAT overexpression under low glucose conditions. **D-E)** Top 10 proteomic pathways **(D)** upregulated or **(E)** downregulated with CrAT overexpression under low glucose conditions. **(F)** Proteomic pathways of interest that were downregulated with CrAT overexpression under low glucose conditions. **(G)** LogFC protein expression and **(H)** individual aceylation of expression of Acetyltransferase and deacetylases with CrAT overexpression under different glucose conditions. Heatmap comparing top **(I)** enriched canonical pathways and **(J)** upstream regulators (by z-score) for global protoeomics and acetylation events with CrAT overexpression under different glucose conditions.

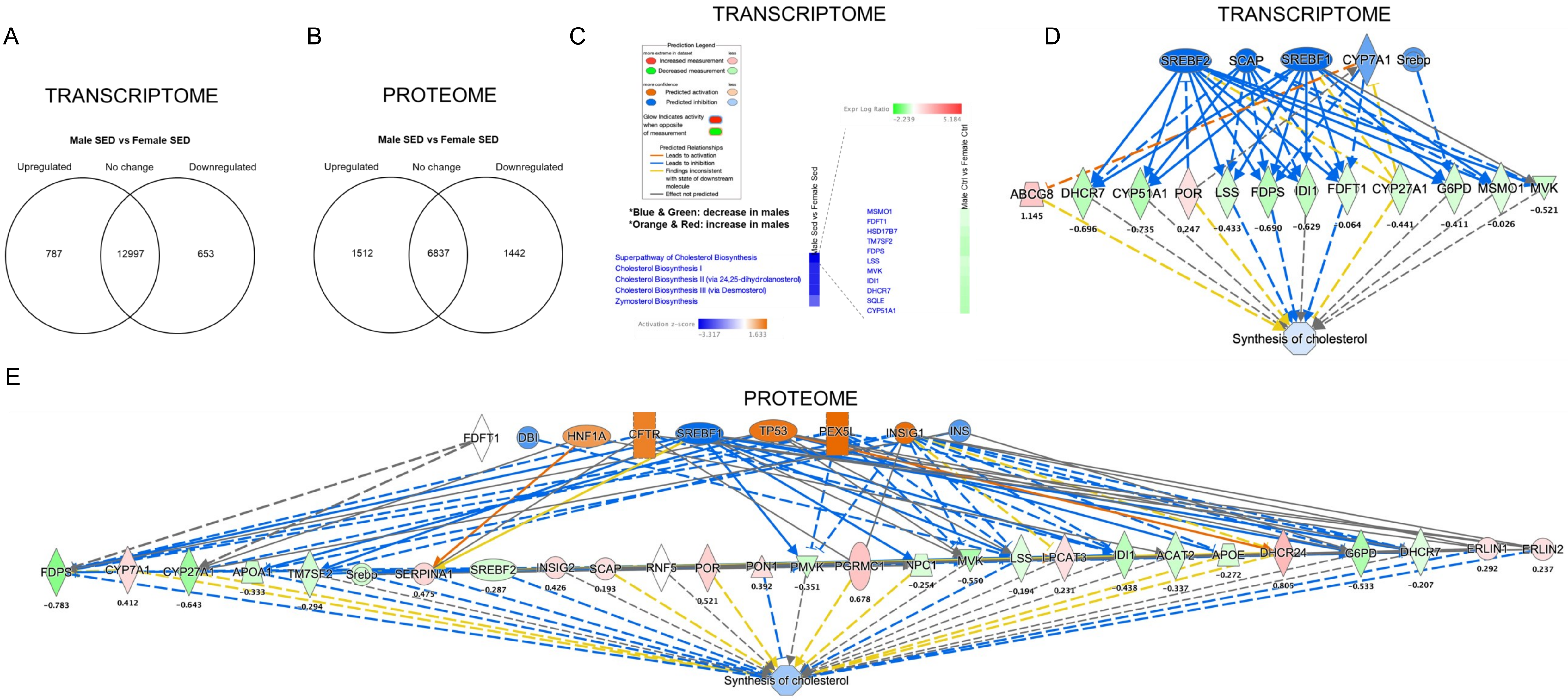
A
TRANSCRIPTOME
Male SED vs Female SED
Upregulated
No change
Downregulated
787
12997
653
B
PROTEOME
Male SED vs Female SED
Upregulated
No change
Downregulated
1512
6837
1442
C
TRANSCRIPTOME
Prediction Legend
Increased measurement
Decreased measurement
Predicted activation
Predicted inhibition
Glow indicates activity when opposite of measurement
Predicted Relationships
Leads to activation
Leads to inhibition
Findings inconsistent with state of downstream molecule
Effect not predicted
*Blue & Green: decrease in males
*Orange & Red: increase in males
Superpathway of Cholesterol Biosynthesis
Cholesterol Biosynthesis I
Cholesterol Biosynthesis II (via 24,25-dihydrolanosterol)
Cholesterol Biosynthesis III (via Desmosterol)
Zymosterol Biosynthesis
Activation z-score
-3.317
1.633
Male Sed vs Female Sed
Expr Log Ratio
-2.239
5.184
Male Ctrl vs Female Ctrl
MSMO1
FDFT1
HSD17B7
TM7SF2
FDPS
LSS
MVK
IDI1
DHCR7
SQLE
CYP51A1
D
TRANSCRIPTOME
SREBF2
SCAP
SREBF1
CYP7A1
Srebp
ABCG8 1.145
DHCR7 -0.696
CYP51A1 -0.735
POR 0.247
LSS -0.433
FDPS -0.690
IDI1 -0.629
FDFT1 -0.064
CYP27A1 -0.441
G6PD -0.411
MSMO1 -0.026
MVK -0.521
Synthesis of cholesterol
E
PROTEOME
FDFT1
DBI
HNF1A
CFTR
SREBF1
TP53
PEX5L
INSIG1
INS
FDPS -0.783
CYP7A1 0.412
CYP27A1 -0.643
APOA1 -0.333
TM7SF2 -0.294
Srebp
SERPINA1 0.475
SREBF2 -0.287
INSIG2 0.426
SCAP 0.193
RNF5
POR 0.521
PON1 0.392
PMVK -0.351
PGRMC1 0.678
NPC1 -0.254
MVK -0.550
LSS -0.194
LPCAT3 0.231
IDI1 -0.438
ACAT2 -0.337
APOE -0.272
DHCR24 0.805
G6PD -0.533
DHCR7 -0.207
ERLIN1 0.292
ERLIN2 0.237
Synthesis of cholesterol

**Figure S3: Transcriptome and proteome predicted reduced cholesterol biosynthesis in the liver of male compared to female sedentary rats. A and B)** Venn diagram of differentially expressed genes (DEGs) and differentially abundant proteins (DAPs) in male verse female sedentary rats. **C)** Comparison analysis heatmaps of top regulated biological functions from IPA transcriptomic analysis in male verse female sedentary rats (bottom left panel) and genes associated with super pathway of cholesterol biosynthesis (right panel). IPA figure legend (upper left panel). **D and E)** Transcriptomic and proteomic activated upstream regulators predicted by IPA display downregulated cholesterol synthesis in male versus female sedentary rats. DEGs and DAPs take all timepoints and sexes into account (FDR<0.05). Blue and green colors represent a decrease and orange and red colors represent an increase in males compared to female sedentary rats. DEGs and DAPs are shown below each node and displayed as $Log_2FC$. For DEGs and DAPs, a z-score ≥ 2 was considered activated.

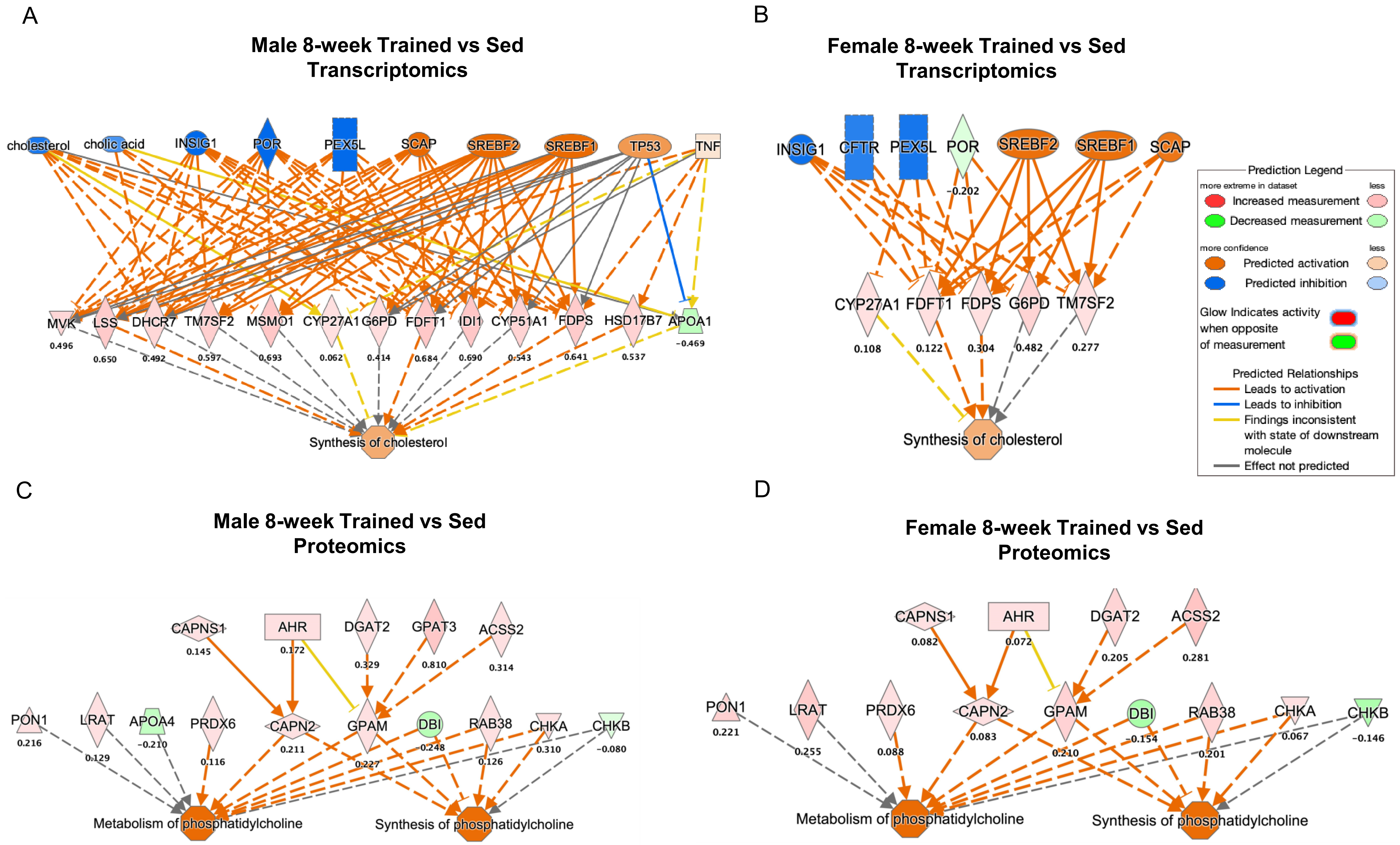
A
Male 8-week Trained vs Sed
Transcriptomics
cholesterol
cholic acid
INSIG1
POR
PEX5L
SCAP
SREBF2
SREBF1
TP53
TNF
MVK
0.496
LSS
0.650
DHCR7
0.492
TM7SF2
0.597
MSMO1
0.693
CYP27A1
0.062
G6PD
0.414
FDFT1
0.684
IDI1
0.690
CYP51A1
0.543
FDPS
0.641
HSD17B7
0.537
APOA1
−0.469
Synthesis of cholesterol
B
Female 8-week Trained vs Sed
Transcriptomics
INSIG1
CFTR
PEX5L
POR
−0.202
SREBF2
SREBF1
SCAP
CYP27A1
0.108
FDFT1
0.122
FDPS
0.304
G6PD
0.482
TM7SF2
0.277
Synthesis of cholesterol
Prediction Legend
more extreme in dataset
less
Increased measurement
Decreased measurement
more confidence
less
Predicted activation
Predicted inhibition
Glow Indicates activity when opposite of measurement
Predicted Relationships
Leads to activation
Leads to inhibition
Findings inconsistent with state of downstream molecule
Effect not predicted
C
Male 8-week Trained vs Sed
Proteomics
CAPNS1
0.145
AHR
0.172
DGAT2
0.329
GPAT3
0.810
ACSS2
0.314
PON1
0.216
LRAT
0.129
APOA4
−0.210
PRDX6
0.116
CAPN2
0.211
GPAM
0.227
DBI
−0.248
RAB38
0.126
CHKA
0.310
CHKB
−0.080
Metabolism of phosphatidylcholine
Synthesis of phosphatidylcholine
D
Female 8-week Trained vs Sed
Proteomics
CAPNS1
0.082
AHR
0.072
DGAT2
0.205
ACSS2
0.281
PON1
0.221
LRAT
0.255
PRDX6
0.088
CAPN2
0.083
GPAM
0.210
DBI
−0.154
RAB38
0.201
CHKA
0.067
CHKB
−0.146
Metabolism of phosphatidylcholine
Synthesis of phosphatidylcholine

**Figure S4: Transcriptome and proteome predicted enhanced lipid metabolism after 8 weeks of exercise in the liver, independent of sex.**

**A and B)** Predicted increased synthesis of cholesterol in the liver of **(A)** males and **(B)** females after 8 weeks of treadmill running. **C and D)** Predicted increased synthesis of metabolism/synthesis of phosphatidylcholine in the liver of **(A)** males and **(B)** females after 8 weeks of treadmill running. DEGs take all timepoints and sexes into account (FDR<0.05). Blue and green colors represent a decrease and orange, and red colors represent an increase in endurance training compared to sedentary rats (n=5). Expression levels of DEGs are shown below each node and displayed as $log_2$FC (fold change). DEGs with z-score ≥ 2 were considered activated.

| ug lipid/ ml plasma | F0 | F1 | F2 | F4 | F8 | M0 | M1 | M2 | M4 | M8 |
|---|---|---|---|---|---|---|---|---|---|---|
| Cholesterol | 0.4864 ± 3.383e-002 | 0.2992 ± 4.283e-002* | 0.3568 ± 5.837e-002 | 0.4058 ± 2.496e-002 | 0.3706 ± 3.463e-002 | 0.151 ± 1.339e-002 | 0.176 ± 3.134e-002 | 0.1508 ± 1.631e-002 | 0.1576 ± 8.710e-004 | 0.1336 ± 1.087e-002 |
| PE | 2.092 ± 0.2103 | 1.396 ± 6.713e-002 | 1.429 ± 0.2063 | 1.622 ± 0.2177 | 1.79 ± 0.1940 | 0.1063 ± 2.416e-002 | 0.04164 ± 3.241e-004 | 0.08806 ± 1.616e-002 | 0.111 ± 2.029e-002 | 0.08458 ± 1.387e-002 |
| O-PE | 17.06 ± 0.7284 | 13.74 ± 0.6088 | 15.92 ± 1.009 | 14.77 ± 1.494 | 13.08 ± 0.6880 | 9.606 ± 0.7480 | 8.018 ± 0.4851 | 9.768 ± 0.8472 | 9.062 ± 0.7277 | 8.226 ± 0.7891 |
| PC | 323 ± 15.19 | 265.2 ± 4.443 | 272.2 ± 16.74 | 284.2 ± 14.81 | 285.2 ± 15.12 | 215 ± 5.109 | 226.2 ± 10.44 | 224.6 ± 10.40 | 215.2 ± 6.606 | 218.8 ± 8.760 |
| O-PC | 6.12 ± 0.2987 | 5.186 ± 0.2547 | 5.94 ± 0.3930 | 5.396 ± 0.5073 | 4.724 ± 0.1132 | 3.312 ± 0.1629 | 3.168 ± 0.1818 | 3.54 ± 0.2426 | 3.026 ± 0.1581 | 2.87 ± 0.2894 |
| Carnitine | 1.932 ± 9.367e-002 | 1.814 ± 0.1007 | 2.028 ± 0.1016 | 1.656 ± 9.474e-002 | 1.624 ± 0.1304 | 1.932 ± 9.367e-002 | 1.814 ± 0.1007 | 2.028 ± 0.1016 | 1.656 ± 9.474e-002 | 1.624 ± 0.1304 |
| Hydroxy Cer | 45.66 ± 4.528 | 33.28 ± 2.389 | 41.48 ± 2.827 | 35.54 ± 7.189 | 33.5 ± 5.702 | 46 ± 6.723 | 32.7 ± 6.723e-003 | 41.76 ± 6.723 | 49.52 ± 6.723 | 42.78 ± 6.723 |
| Non-hydroxy Cer | 11.37 ± 0.8720 | 8.694 ± 0.5886 | 10.6 ± 0.5718 | 9.784 ± 1.252 | 9.918 ± 0.6497 | 6.32 ± 0.3065 | 6.484 ± 0.5100 | 7.01 ± 0.5924 | 6.046 ± 0.2374 | 6.88 ± 0.4939 |
| Hex Cer | 2.324 ± 0.2275 | 1.796 ± 8.524e-002 | 2.092 ± 0.1803 | 1.996 ± 0.2044 | 2.048 ± 0.1801 | 0.6156 ± 4.359e-002 | 0.6298 ± 4.999e-002 | 0.626 ± 2.585e-002 | 0.594 ± 2.286e-002 | 0.6126 ± 4.588e-002 |
| Non-hydoxy FA | 4.027 ± 0.4217 | 2.937 ± 0.2106 | 2.624 ± 0.2224 | 3.158 ± 0.5268 | 3.26 ± 0.4249 | 2.958 ± 0.1457 | 3.921 ± 0.3615 | 3.941 ± 0.4404 | 3.726 ± 0.5298 | 2.568 ± 0.2665 |
| DAG | 1.834 ± 0.1687 | 1.338 ± 4.488e-002* | 1.218 ± 6.748e-002** | 1.262 ± 9.856e-002** | 1.74 ± 0.1172 | 2.854 ± 0.2118 | 2.164 ± 0.0001341 | 1.99 ± 0.2614 | 2.864 ± 0.3029 | 2.12 ± 0.2549 |
| TAG | 51.8 ± 5.250 | 33.88 ± 1.878* | 30.96 ± 3.636** | 35.18 ± 3.253* | 49.66 ± 4.927 | 116.4 ± 9.551 | 88.34 ± 6.787 | 75.2 ± 9.847 | 129.5 ± 1.809 | 88.08 ± 13.48 |

**Table S1.** Plasma lipid concentrations in sedentary and trained male and female rats. Significant protein abundance change compared to sedentary conditions within sex is presented by *, $p<0.05$; **, $p<0.01$. Values are represented as mean ± standard error.
